# The m^6^A demethylase ALKBH5-mediated upregulation of DDIT4-AS1 maintains pancreatic cancer stemness and suppresses chemosensitivity by activating the mTOR pathway

**DOI:** 10.1186/s12943-022-01647-0

**Published:** 2022-09-02

**Authors:** Yi Zhang, Xiaomeng Liu, Yan Wang, Shihui Lai, Zhiqian Wang, Yudie Yang, Wenhui Liu, Hongquan Wang, Bo Tang

**Affiliations:** 1 Department of Pancreatic Cancer, Tianjin Medical University Cancer Institute and Hospital, National Clinical Research Center for Cancer, Tianjin’s Clinical Research Center for Cancer, Key Laboratory of Cancer Prevention and Therapy, Tianjin, Huanhu West Road, Hexi District, 300060 Tianjin, China; 2 Department of Genaral Surgery, Affiliated Hospital of Xuzhou Medical University, Xuzhou, 221000 Jiangsu People’s Republic of China; 3 Key Laboratory of Basic and Clinical Application Research for Hepatobiliary Diseases of Guangxi, The First Affiliated Hospital of Guangxi Medical University, Nanning, China

**Keywords:** ALKBH5, Stemness, Chemosensitivity, DDIT4-AS1, UPF1

## Abstract

**Background:**

Chemoresistance is a major factor contributing to the poor prognosis of patients with pancreatic cancer, and cancer stemness is one of the most crucial factors associated with chemoresistance and a very promising direction for cancer treatment. However, the exact molecular mechanisms of cancer stemness have not been completely elucidated.

**Methods:**

m^6^A-RNA immunoprecipitation and sequencing were used to screen m^6^A-related mRNAs and lncRNAs. qRT-PCR and FISH were utilized to analyse DDIT4-AS1 expression. Spheroid formation, colony formation, Western blot and flow cytometry assays were performed to analyse the cancer stemness and chemosensitivity of PDAC cells. Xenograft experiments were conducted to analyse the tumour formation ratio and growth in vivo. RNA sequencing, Western blot and bioinformatics analyses were used to identify the downstream pathway of DDIT4-AS1. IP, RIP and RNA pulldown assays were performed to test the interaction between DDIT4-AS1, DDIT4 and UPF1. Patient-derived xenograft (PDX) mouse models were generated to evaluate chemosensitivities to GEM.

**Results:**

DDIT4-AS1 was identified as one of the downstream targets of ALKBH5, and recruitment of HuR onto m^6^A-modified sites is essential for DDIT4-AS1 stabilization. DDIT4-AS1 was upregulated in PDAC and positively correlated with a poor prognosis. DDIT4-AS1 silencing inhibited stemness and enhanced chemosensitivity to GEM (Gemcitabine). Mechanistically, DDIT4-AS1 promoted the phosphorylation of UPF1 by preventing the binding of SMG5 and PP2A to UPF1, which decreased the stability of the DDIT4 mRNA and activated the mTOR pathway. Furthermore, suppression of DDIT4-AS1 in a PDX-derived model enhanced the antitumour effects of GEM on PDAC.

**Conclusions:**

The ALKBH5-mediated m^6^A modification led to DDIT4-AS1 overexpression in PDAC, and DDIT-AS1 increased cancer stemness and suppressed chemosensitivity to GEM by destabilizing DDIT4 and activating the mTOR pathway. Approaches targeting DDIT4-AS1 and its pathway may be an effective strategy for the treatment of chemoresistance in PDAC.

**Supplementary Information:**

The online version contains supplementary material available at 10.1186/s12943-022-01647-0.

## Background

Pancreatic cancer is a prevalent gastrointestinal malignancy worldwide and is characterized by a late diagnosis, poor prognosis and high mortality. Pancreatic cancer is currently the 4th leading cause of cancer-related death, with a 5-year survival rate of only approximately 10% [1]. Pancreatic ductal adenocarcinoma (PDAC) is the most common type of pancreatic cancer. Gemcitabine (GEM) is the first-line chemotherapeutic agent for pancreatic cancer, and adjuvant chemotherapy with GEM has been shown to significantly prolong the overall survival and disease-free survival of patients with pancreatic cancer [2]. However, the resistance of malignant tumour cells to GEM remarkably affects the efficacy of treatment. Cancer stem cells (CSCs) are an important factor contributing to chemotherapy resistance in pancreatic cancer [3]. CSCs have self-renewal and multilineage differentiation abilities, which are associated with drug resistance, recurrence, and metastasis of malignant tumours [4]. Nevertheless, the mechanisms by which CSCs affect the progression and drug resistance of pancreatic cancer remain obscure and require further exploration.

N^6^-methyladenosine (m^6^A) mRNA modification is a highly prevalent in RNA and plays an important role in cancer initiation and progression [5, 6]. The m^6^A modification mediates mRNA stability and translation efficiency [7]. This modification is installed by the writers METTL3 and METTL14 and can be removed by the erasers FTO and ALKBH5 [8]. ALKBH5 belongs to the AlkB family of nonheme Fe(II)/a-ketoglutarate-dependent dioxygenases. ALKBH5 was reported to mediate the hypoxia-induced and hypoxia-inducible factor (HIF)-dependent breast CSC phenotype [9]. Furthermore, our previous studies reported the downregulation of ALKBH5 in a GEM-treated patient-derived xenograft (PDX) model, and its overexpression sensitizes PDAC cells to chemotherapy [10], suggesting its potential roles in regulating stemness and chemosensitivity. However, the regulatory mechanisms of ALKBH5 remain largely unknown.

DNA damage-inducible transcript 4 (DDIT4), also called DNA damage response 1 (REDD1) and stress-triggered protein (RTP801), consists of 232 amino acids and is mainly found in the cytoplasm and nucleus [11, 12]. Upon stress induction, including oxidative stress, endoplasmic reticulum stress, hypoxia and starvation [11, 13–15], DDIT4 inhibits mTOR signalling by stabilizing the tuberous sclerosis complex (TSC1-TSC2) [16, 17], DDIT4 is aberrantly expressed at low levels in multiple tumours, including gastric cancer [18], breast cancer [19], glioma [20] and other tumours [21–23] The antisense transcript of DDIT4 encodes the lncRNA DDIT4-AS1, which has a length of 847 bp and consists of two exons. Numerous studies have indicated that abnormal expression of antisense lncRNAs contributes to tumorigenesis, oncogenic progression and the hallmarks of cancer [24]. However, the function of DDIT4-AS1 has not been previously reported.

In this study, we identified DDIT4-AS1 as a downstream target of ALKBH5, and recruitment of HuR onto m^6^A-modified sites is essential for DDIT4-AS1 stabilization. We also described the function of DDIT4-AS1 in maintaining pancreatic cancer stemness and suppressing chemosensitivity. Mechanistically, DDIT4-AS1 recruits UPF1 to destabilize DDIT4 and activate the mTOR pathway, suggesting the crucial roles of DDIT4-AS1 in cancer progression.

## Materials and methods

### Patient samples

This study included 40 pairs of fresh PDAC tissues obtained from the First Affiliated Hospital of Guangxi Medical University (GXMU) that were stored at − 80 °C immediately after surgery. We obtained informed consent from all patients. The study was approved by the Ethics Committee of the Provincial Clinical College of GXMU based on the ethical guidelines of the 1975 Declaration of Helsinki. The clinicopathological characteristics examined included age, sex, tumour nodule number, aetiology, tumour stage, tumour size, tumour differentiation, and vascular invasion. The tumour classification and grade were determined based on the TNM classification.

### Chemicals and antibodies

Lipofectamine 2000 transfection reagents and total RNA extraction agent (TRIzol) were purchased from Invitrogen (Grand Island, NY, USA). Antibodies against Ki67, DDIT4, ALKBH5, Mettle3, HuR, Caspase-3, mTOR, p-mTOR, p-ULK, p-Ps70S6K, CD133, and CD44 were obtained from Abcam (Cambridge, MA, USA), whereas those against OCT4, SOX2, and EpCAM were obtained from Cell Signaling Technology (Danvers, MA, USA). UPF1 antibodies were purchased from Becton Dickinson. Unless indicated otherwise, all other chemicals were purchased from Sigma-Aldrich (St. Louis, MO, USA). Details of antibody information are provided in Supplementary materials.

### Cell lines and cell culture

The human cell lines 293 T, AsPC-1, PANC-1, BxPC-3, HPDE6-C7, CFPAC-1, and SW1990 were purchased from American Type Culture Collection (ATCC). Cells were cultured in DMEM (Gibco, Grand Island, New York, USA) supplemented with 10% foetal bovine serum (FBS, Gibco), 1% penicillin and 1% streptomycin and incubated in an incubator with 5% CO_2_ at 37 °C.

### Quantitative real-time polymerase chain reaction (qRT-PCR)

Total RNA was extracted using TRIzol reagent (Invitrogen), and complementary DNA (cDNA) templates were synthesized using SuperScript II Reverse Transcriptase (Invitrogen). Quantitative reverse transcription-PCR (qRT-PCR) and data collection were performed with an ABI PRISM 7900HT sequence detection system.

Total RNA was extracted from selected cells or tissues using TRIzol reagent (Invitrogen, Carlsbad, CA, USA) according to the manufacturer’s instructions. Complementary DNA (cDNA) templates were synthesized using SuperScript II Reverse Transcriptase (Invitrogen). Fast Start Universal SYBR Green Master Mix (Roche Diagnostics GmbH, Mannheim, Germany) was used for qRT-PCR. The primers used to detect mRNA levels are listed in Supplementary materials.

### Immunohistochemistry (IHC)

IHC was performed on paraformaldehyde-fixed, paraffin-embedded tissue specimens following heat-mediated antigen retrieval in citrate buffer (0.01 M, pH 6.0). Endogenous peroxidase activity was blocked with 3% H_2_O_2_ for 15 min at room temperature (RT). Thereafter, the sections were incubated with goat serum for 1 h to block the nonspecific binding sites and then with primary antibodies overnight at 4 °C. After three rinses with PBS for 5 min each, the sections were further incubated with horseradish peroxidase-conjugated secondary antibodies for 1 h at RT. Each section was then rinsed with PBS three times for 5 min each, and the reactions were developed using diaminobenzidine tetrahydrochloride (DAB) as a substrate. Cellular nuclei were counterstained using haematoxylin, and the sections were sealed with neutral gum. Images were obtained using an Olympus X71 inverted microscope (Olympus Corp., Tokyo, Japan).

### Plate colony-forming and sphere-forming assays

For the plate colony-forming assay, PC cells were plated into 6-well plates at a density of 5 × 10^2^ cells per well and cultured for 14 days. After the incubation, the cells were rinsed with PBS, fixed with methanol/acetic acid (3:1, v/v), and then stained with 0.5% crystal violet (Sigma-Aldrich, St. Louis, MO, USA). Mammosphere culture was performed as described by Dontu et al. Single-cell suspensions were cultured in 6-well plates (Costar) at different densities of viable cells with serum-free epithelial growth medium supplemented with a 1:50 dilution of B27 (Invitrogen), 20 ng/ml epidermal growth factor, 20 ng/ml basic fibroblast growth factor (BD), and 10 μg/ml heparin (Sigma). The number of spheroids was counted after 2 weeks. The resulting cells were counted using an Olympus IX81 high-resolution microscope (Olympus).

### Flow cytometry

The cells were suspended to a density of 1 × 10^6^ cells/ml, and 5 μL of Annexin V and propidium iodide (PI) staining solution were added to 300 μL of the cell suspension. After an incubation for 10–15 min at room temperature in the dark, stained cells were assayed and quantified using a FACSort Flow Cytometer (BD, San Jose, CA, USA). Each experiment was performed in triplicate and repeated at least twice.

### Western blotting

Protein samples were extracted using cold RIPA lysis buffer containing protease inhibitor cocktail (Roche, Mannheim, Germany), and the protein concentration was determined using the BCA method. Equal amounts of protein lysates were separated by sodium dodecyl sulfate polyacrylamide gel electrophoresis (SDS-PAGE) using a Bio-Rad system. Separated proteins were detected with the corresponding antibodies and visualized using enhanced chemiluminescence (ECL).

### Coimmunoprecipitation

Cells were collected in cold PBS and lysed with immunoprecipitation (IP) lysis buffer (25 mM Tris•HCl, pH 7.4, 150 mM NaCl, 1% NP-40, 1 mM EDTA, and 5% glycerol). Then, 2–5 μg of antibodies were added to the diluted cell lysates, and the mixtures were incubated overnight at 4 °C. The next day, the protein complexes were isolated with magnetic Protein A/G Dynabeads for 2 h at 4 °C with rotation. The bead-antibody-protein complexes were then washed 4 times with wash buffer (50 mM Tris (pH 7.4), 125 mM NaCl, 1 mM EDTA and 0.1% NP-40) and boiled before Western blot analysis.

### Generation of the subcutaneous xenograft model in mice and establishment of PDX

All animal experimental protocols were performed in accordance with the National Institutes of Health Guidelines for the Care and Use of Experimental Animals.

Four-week-old BALB/c nude mice were purchased from the Animal Center of Guangxi Medical University (Nanning, China). For this experiment, 2 × 10^6^ PDAC cells transfected with shDDIT4-AS1 or the negative control were subcutaneously injected into the flank region of the mice. The tumour volume was measured weekly and calculated as follows: V = (length × width^2^)/2. After four weeks, the tumours were excised and weighed.

Fresh tumor fragments were transplanted subcutaneously (s.c.) into the left flank of anaesthetized NOD scid gamma mice. Mice were observed for maximum 120 d and maintained under sterile and controlled conditions (22 °C, 50% relative humidity, 12 h light–dark cycle, autoclaved food and bedding, acidified drinking water). Tumor growth was measured in 2 dimensions with a caliper. Tumor volumes (TV) were determined by the formula: TV = (length × width^2^)/2. Tumors were routinely passaged at TV = 1 cm^3^. Xenograft material was snap frozen and stored at -80 °C or processed to formalin fixed, paraffin embedded (FFPE) blocks.

### Fluorescence in situ hybridization (FISH)

PDAC cells grew on glass cover slips in 24-well plates overnight. The cells were fixed with paraformaldehyde and hybridized with DDIT4-AS probe was used to stain the nuclei. The results were observed under confocal microscopy.

### Immunofluorescence (IF)

The cells were washed with PBS (Beyotime, C0221A) and then fixed with 4% paraformaldehyde (Sigma, V900894) for 30 min at room temperature. After being washed 3 times with PBS (Beyotime, C0221A), the cells were blocked in 10% donkey serum (Solarbio, S9100) for 10 min. The cells were subsequently incubated with primary antibodies specific overnight at 4 °C. The next day, the cells were washed with PBS, after which they were incubated with fluorescent-conjugated secondary antibodies (Beyotime, A0562), followed by DAPI (Beyotime, C1002). Images were captured by confocal microscopy.

### DDIT4-AS1 RNA or its fragments and the UPF1 cDNA fragments

The full‐length sense and antisense of DDIT4-AS1 RNA or its fragments (1–250, 251–500, 501–700, 401–847) were in vitro transcribed by using MEGAscript™ T7 Transcription Kit (Thermo Fisher Scientific, Waltham, MA, USA). The synthesized RNA probes were confirmed by RNA sequencing (RiboBio, Guangzhou, China).

Fragments encoding UPF1 amino acid residues were generated by PCR and inserted into a pcDNA3.1-Flag vector, which was synthesized by GenePharma (Shanghai, China). The details of PCR primers are listed in Supplementary materials.

### Microarray analysis

We performed a microarray analysis using PDAC cells transfected with shDDIT4-AS1 or its negative control. Total RNA was extracted using an RNA simple Total RNA Kit (TIANGEN, DP419), and a NanoDrop One spectrophotometer (Thermo Fisher Scientific) was used to measure the RNA quantity. The microarray analysis was performed by Huayin Health Medical Group Co., Ltd. (Guangzhou, China). The obtained data were then subjected to KEGG and GO analyses. Cluster 3.0 and Java Tree View software were used to visualize heat maps.

### RNA immunoprecipitation (RIP)

RIP was conducted with the Magna RIP RNA-Binding Protein Immunoprecipitation Kit (Millipore, USA) according to the manufacturer’s instructions. Briefly, magnetic beads coated with 5 μg of specific antibodies were incubated with prepared cell lysates overnight at 4 °C. Then, the RNA–protein complexes were washed 6 times and incubated with proteinase K digestion buffer. RNA was finally extracted using phenol–chloroform RNA extraction methods. The relative RNA expression was determined using qPCR and normalized to the input.

### Methylated RNA immunoprecipitation qPCR (MeRIP-qPCR)

The MeRIP-qPCR assay was performed to determine the level of m^6^A. Total intracellular RNA was extracted using TRIzol reagent. Anti-m^6^A antibodies or 9 anti-immunoglobulin G (IgG; Cell Signaling Technology) (3 μg) was first conjugated to Protein A/G magnetic beads and mixed with a 100 μg aliquot of total RNA in IP buffer containing RNase/protease inhibitors. The m^6^A-modified RNA was eluted twice with 6.7 mM N^6^-methyladenosine 5'-monophosphate sodium salt at 4 °C for 1 h. Subsequently, RT-qPCR analysis was performed to determine m^6^A enrichment. The methylated RNA was purified for further MeRIP sequencing by RiboBio (Guangzhou, China).

### Northern blotting

Total RNA of BMDMs was extracted using TRI reagent (Sigma, USA). Biotin-labeled antisense and sense RNA probes (300 bp, 1,441 to 1,740 nt) were made in vitro using a HiScribe T7 Quick high-yield RNA synthesis kit (NEB, United Kingdom) and biotin RNA labeling mix (Roche, Germany). The assay was carried out according to the manufacturer’s protocol (NorthernMax kit; Thermo Fisher, USA).

### RNA pull-down assay

A Pierce Magnetic RNA–Protein Pull-Down Kit (Thermo Fisher Scientific, 20,164) was used according to the manufacturer’s instructions. In brief, cell lysates were treated with RNAase-free DNAase I and incubated with biotinylated DDIT4-AS1 in the presence of streptavidin magnetic beads, which can capture the proteins/miRNAs potentially interacting with DDIT4-AS1 A Pierce™ RNA 3' End Desthiobiotinylation Kit (Thermo, 20,163) was used for DDIT4-AS1 biotinylation labelling. Proteins and RNAs in the captured protein-RNA.

### RNA stability

For the transcriptional inhibitor actinomycin D (Millipore, Billerica, MA, USA). Cells were incubated with 10ug/mL actinomycin D at various time points. Real-time PCR was then performed the relative abundance of each mRNA. The relative amount of mRNA at 0 h following actinomycin D treatment was arbitrarily set to 1 h.

### Detection of CD133 expression using flow cytometry

CD133^+^ cells were sorted by FACS with Anti-CD133/1-phycoerythrin (eBioscience, USA). Only the top 2%, corresponding to the most brightly stained cells or the bottom 2% corresponding to the most dimly stained cells were selected as CD133 positive and negative populations, respectively.

### Statistical analysis

Statistical analyses were performed using SPSS 22.0 software. Comparisons between different groups were calculated using Student’s t-test, one-way ANOVA, or the Mann–Whitney U test. The clinical prognosis was assessed using the Kaplan–Meier analysis. Pearson’s correlation analysis was performed to determine correlations. Statistical significance was set to *p* < 0.05. Data were recorded as means ± SD.

## Results

### DDIT4-AS1 was regulated by ALKBH5 in an m^6^A-dependent manner

**Fig. 1:**
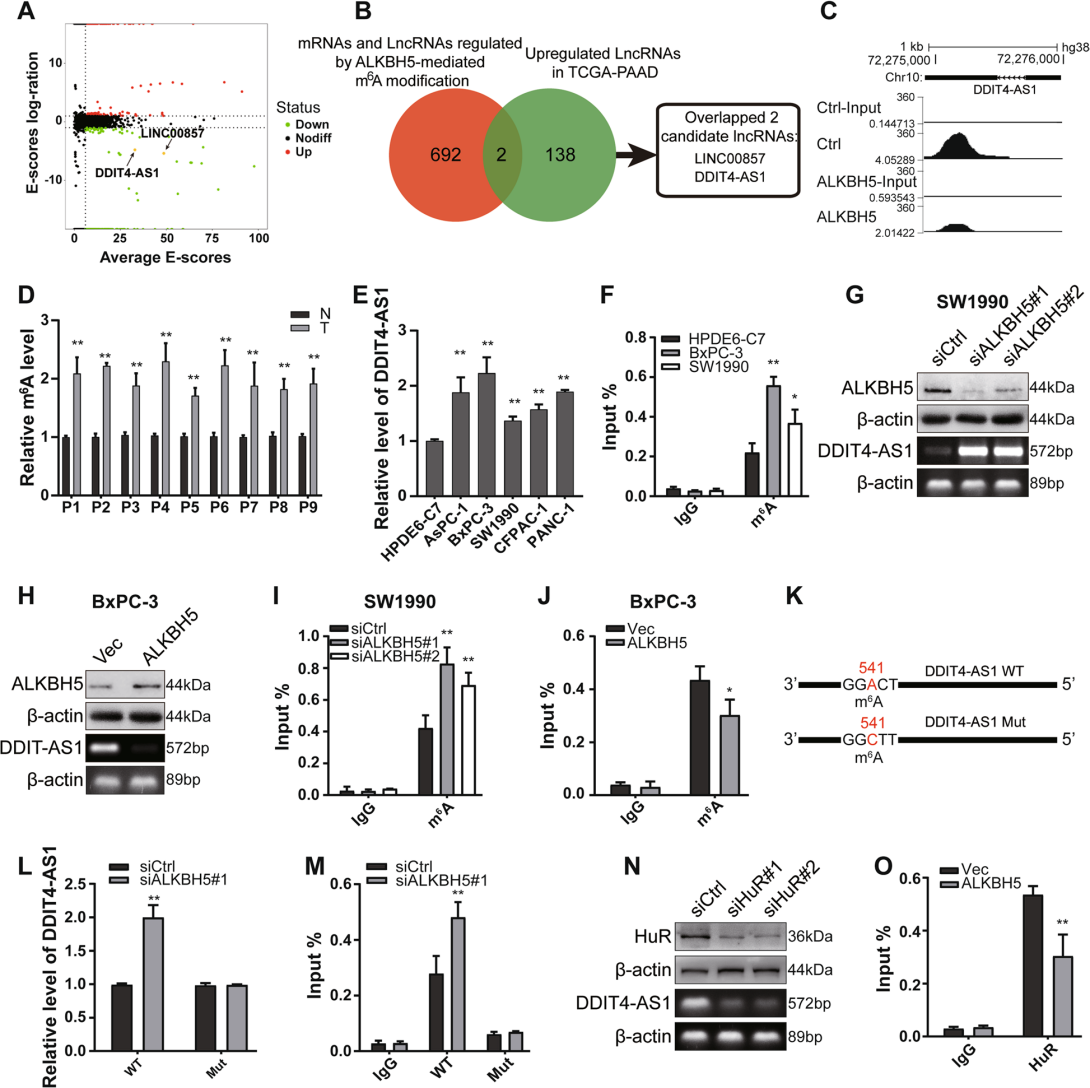
DDIT4-AS1 was regulated by ALKBH5 in an m^6^A-dependent manner. **a** Volcano plot showing the differences in peaks between ctrl- and ALKBH5-overexpressing PDAC cells. Average E-score ≥ 6, |Log2(ctrl E-score/experimental E-score) |≥ 1. **b** Venn diagram showing the numbers of overlapping lncRNAs between the differentially upregulated lncRNAs in TCGA-PDAC and the differentially expressed lncRNAs and mRNAs after ALKBH5 overexpression. **c** MeRIP-seq data showed the distribution of m^6^A peaks along DDIT4-AS1 in BxPC-3 cells transfected with the ALKBH5 plasmid compared to those transfected with the vector. **d** The m^6^A levels of DDIT4-AS1 from human PDAC tissues and normal adjacent tissues determined using MeRIP-qPCR. **e** The RNA levels of DDIT4-AS1 from PDAC cell lines and a pancreatic duct epithelial cell line (HPDE6-C7) were determined using qPCR. **f** MeRIP-qPCR analysis of m^6^A enrichment of DDIT4-AS1 in PDAC cell lines and pancreatic duct epithelial cell lines. **g-h** WB and RT-PCR analyses of ALKBH5 and DDIT4-AS1 levels in SW1990 cells transfected with the ALKBH5 siRNA and BxPC-3 cells transfected with the ALKBH5 plasmid. **i-j** MeRIP-qPCR analysis of the m^6^A levels in DDIT4-AS1 in SW1990 cells transfected with the ALKBH5 siRNA and BxPC-3 cells transfected with the ALKBH5 plasmid. **k** Putative m^6^A modification sites in the coding sequence of DDIT4-AS1 and synonymous mutations in DDIT4-AS1. **l** The relative expression levels of the DDIT4-AS1 RNA in SW1990 cells cotransfected with ALKBH5 siRNAs and DDIT4-AS1-WT or DDIT4-AS1-Mut. **m** MeRIP-qPCR analysis of the m^6^A levels in DDIT4-AS1 in SW1990 cells cotransfected with ALKBH5 siRNAs and DDIT4-AS1-WT or DDIT4-AS1-Mut. **n** WB and RT-PCR analyses of HuR and DDIT4-AS1 levels in BxPC-3 cells transfected with HuR siRNAs. **o** RIP analysis of the interaction of HuR with DDIT4-AS1 in BxPC-3 cells transfected with the ALKBH5 overexpression plasmid compared to cells transfected with the empty vector. Data are presented as the means ± SD of three independent experiments (*, *P* < 0.05; **, *P* < 0.01)

As shown in our previous study, ALKBH5 overexpression sensitizes PDAC cells to chemotherapy [10]. We identified whether lncRNAs were demethylated by ALKBH5 and were involved in the malignant phenotype of PDAC cells by performing MeRIP sequencing in cells with or without ALKBH5 overexpression. The results revealed a significant reduction in m^6^A levels in 425 mRNAs and lncRNAs and a significant increase in m^6^A levels in 269 mRNAs and lncRNAs (Fig. 1a). Then, we screened differentially expressed lncRNAs in the TCGA-PDAC database, and 140 upregulated lncRNAs (LogFC ≥ 1) were identified in patients with PDAC. By comparing the two datasets listed above, an overlap of 2 lncRNAs was identified (Fig. 1b). Among these 2 genes, DDIT4-AS1 has rarely been studied and was selected for subsequent analysis.

Upon ALKBH5 overexpression, we observed a decrease in m^6^A peaks on DDIT4-AS1 based on MeRIP-seq (Fig. 1c). We further investigated whether DDIT4-AS1 was regulated by ALKBH5-mediated m^6^A modification by detecting DDIT4-AS1 expression and its m^6^A level in clinical PDAC samples. DDIT4-AS1 expression and m^6^A levels of DDIT4-AS1 were higher in PDAC tissues than in normal adjacent tissues (Fig. 1d and S1a). Then, we detected DDIT4-AS1 levels in PDAC cell lines (BxPC-3, AsPC-1, SW1990, CFPAC-1 and PANC-1) and a pancreatic ductal epithelial cell line (HPDE6-C7). The expression level of DDIT4-AS1 was upregulated in PDAC cell lines compared to pancreatic ductal epithelial cells (Fig. 1e). A similar result was obtained for the m^6^A analysis in BxPC-3, SW1990 and HPDE6-C7 cells (Fig. 1f). Furthermore, ALKBH5 knockdown increased the level of DDIT4-AS1 and its m^6^A modification in SW1990 cells (Fig. 1g and i); in contrast, ALKBH5 overexpression produced the opposite result in BxPC-3 cells (Fig. 1h and j).

We next searched for the m^6^A consensus motifs using STRAMP (www.cuilab.cn/sramp/) and identified the GGACU sequence at bases 539–543. We constructed Flag-tagged DDIT4-AS1 wt and mut plasmids (Fig. 1k). Depletion of ALKBH5 led to increased expression of wt DDIT4-AS1 but not mut DDIT4-AS1 (Fig. 1l), whereas overexpression of ALKBH5 led to decreased expression of wt DDIT4-AS1 but not mut DDIT4-AS1 (Fig. S1b). Then, we evaluated the level of the m^6^A modification in wt and mut DDIT4-AS1 using MeRIP-qPCR. As expected, the depletion of ALKBH5 increased the m^6^A level of wt DDIT4-AS1 but did not change the m^6^A level of mut DDIT4-AS1 (Fig. 1m). Based on these data, the m^6^A site was essential for ALKBH5-mediated DDIT4-AS1 expression. m^6^A readers, such as HuR and IGF2BP1, have been reported to recognize m^6^A sites and stabilize RNA. We explored how the m^6^A modification affects DDIT4-AS1 expression by knocking down HuR and IGF2BP1 in PDAC cells. Intriguingly, the depletion of HuR, but not IGF2BP1, significantly decreased the expression of DDIT4-AS1 (Fig. 1n, S1c). A RIP assay confirmed the direct interaction between HuR and DDIT4-AS1 in PDAC cells. In addition, the interaction between HuR and DDIT4-AS1 was impaired after ALKBH5 overexpression (Fig. 1o, S1d). Thus, HuR binds to DDIT4-AS1 to increase its expression in an m^6^A-dependent manner.

### DDIT4-AS1 is upregulated in PDAC and predicts shorter survival

**Fig. 2:**
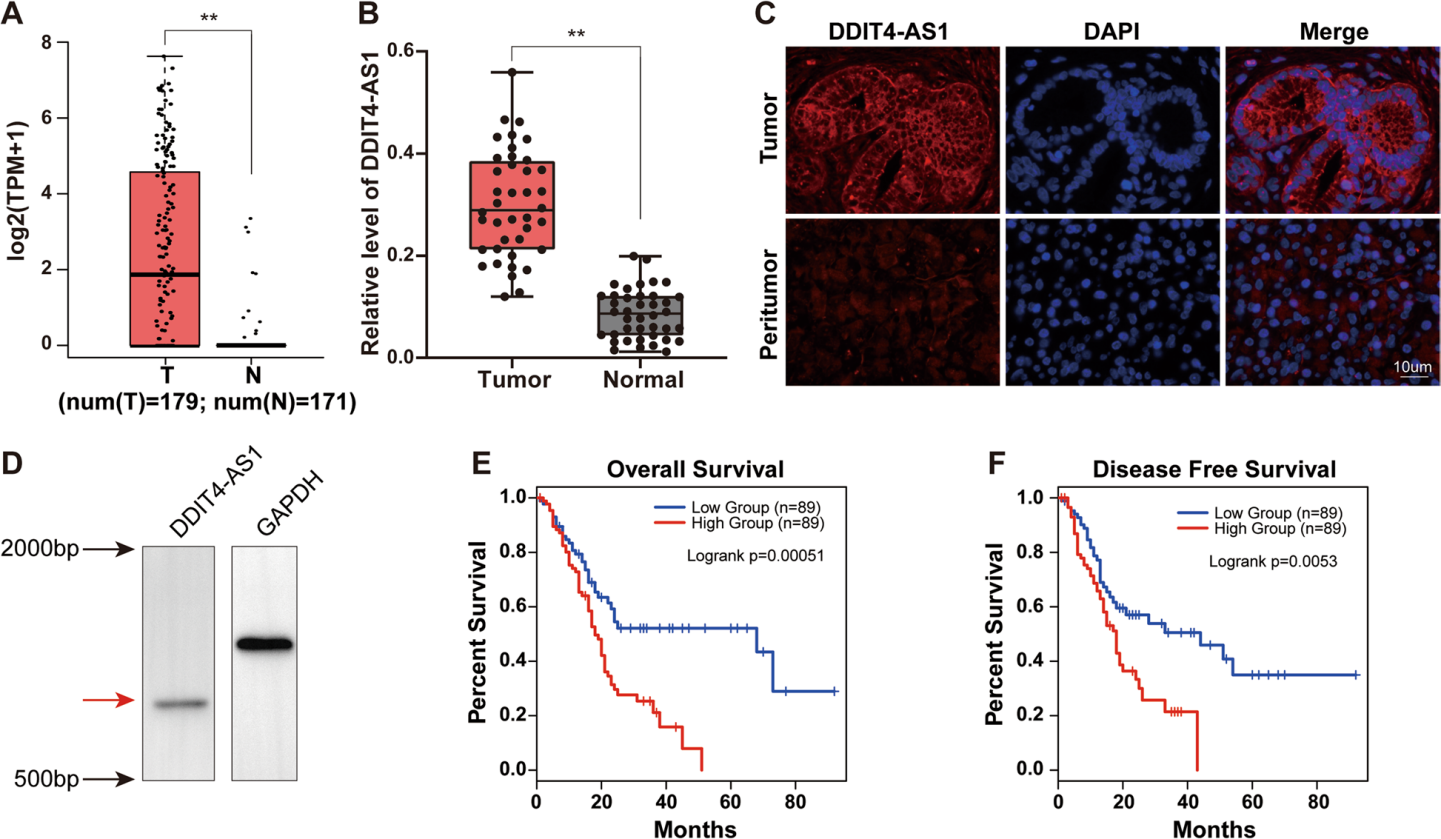
DDIT4-AS1 is upregulated in PDAC and predicts shorter survival. **a** DDIT4-AS1 expression was assessed in PDAC tissues and normal adjacent tissues using data from TCGA. **b** The relative expression levels of DDIT4-AS1 were assessed in 40 paired PDAC tissues and normal adjacent tissues using qRT-PCR. **c** Immunofluorescence staining for DDIT4-AS1 expression in PDAC tissue and normal adjacent tissue. **d** Northern blot analysis of DDIT4-AS1 expression in BxPC-3 cells. **e–f** Kaplan–Meier analysis of the RFS and OS rates in 178 patients with PDAC presenting high or low expression of DDIT4-AS1 using data from TCGA. Data are presented as the means ± SD of three independent experiments (*, *P* < 0.05; **, *P* < 0.01)

We assessed the expression of DDIT4-AS1 in PDAC by analysing the expression of DDIT4-AS1 in TCGA and GTEx databases; the results revealed that DDIT4-AS1 was significantly upregulated in PDAC tissues compared to normal tissues (Fig. 2a). This trend was further verified in randomly selected specimens using qRT-PCR (*n* = 40, Fig. 2b). In addition, the FISH results confirmed that DDIT4-AS1 was mainly located in the cytoplasm and that the level of DDIT4-AS1 was comparatively higher than that in normal tissues (Fig. 2c). Northern blot analysis with a specific RNA probe for DDIT4-AS1 showed that DDIT4-AS1 was detected at its expected size of 847 bp (Fig. 2d).

We evaluated the potential correlation between DDIT4-AS1 expression and clinicopathological features and found that DDIT4-AS1 was positively associated with the tumour size (*p* = 0.022) and tumour stage (*p* = 0.016, Table 1). The Kaplan–Meier analysis revealed that higher DDIT4-AS1 expression was associated with lower overall survival (OS) rates (*p* = 0.0038, Fig. 2e) and lower disease-free survival (DFS) rates for 269 patients with PDAC from TCGA (*p* = 0.026, Fig. 2f).

**Table 1:** Relationship between DDIT4-AS1 and clinicopathological parameters in 70 PADC patients

Variables	All cases	DDIT4-AS1 expression		*HR(95%CI)*	*χ*^*2*^	p*
		Low (*n* = 35)	High (*n* = 35)			
**Age(years)**						
≥ 60	46	25	21	1.667(0.615–4.519)		
60	24	10	14		0.163	0.314
**Gendar**						
Male	38	20	18	1.259(0.491–3.230)		
Female	32	15	17		0.808	0.631
**Tumor size(cm)**						
˂3	23	16	7	3.368(1.164–9.744)		
≥ 3	47	19	28		0.001	0.022
**Tumor differentiation**						
Well/ Moderate	41	18	23	0.552(0.211–1.446)		
Poor	29	17	12		0.082	0.225
**Lymph node invasion**						
Absent	52	28	24	1.833(0.614–5.471)		
Present	18	7	11		0.123	0.274
**TNM stage**						
I-II	32	21	11	3.273(1.224–8.748)		
III-IV	38	14	24		0.000	0.016

^*^ Probability, *P*, from χ^2^ test

### DDIT4-AS1 maintains the stemness properties of PDAC cells

**Fig. 3:**
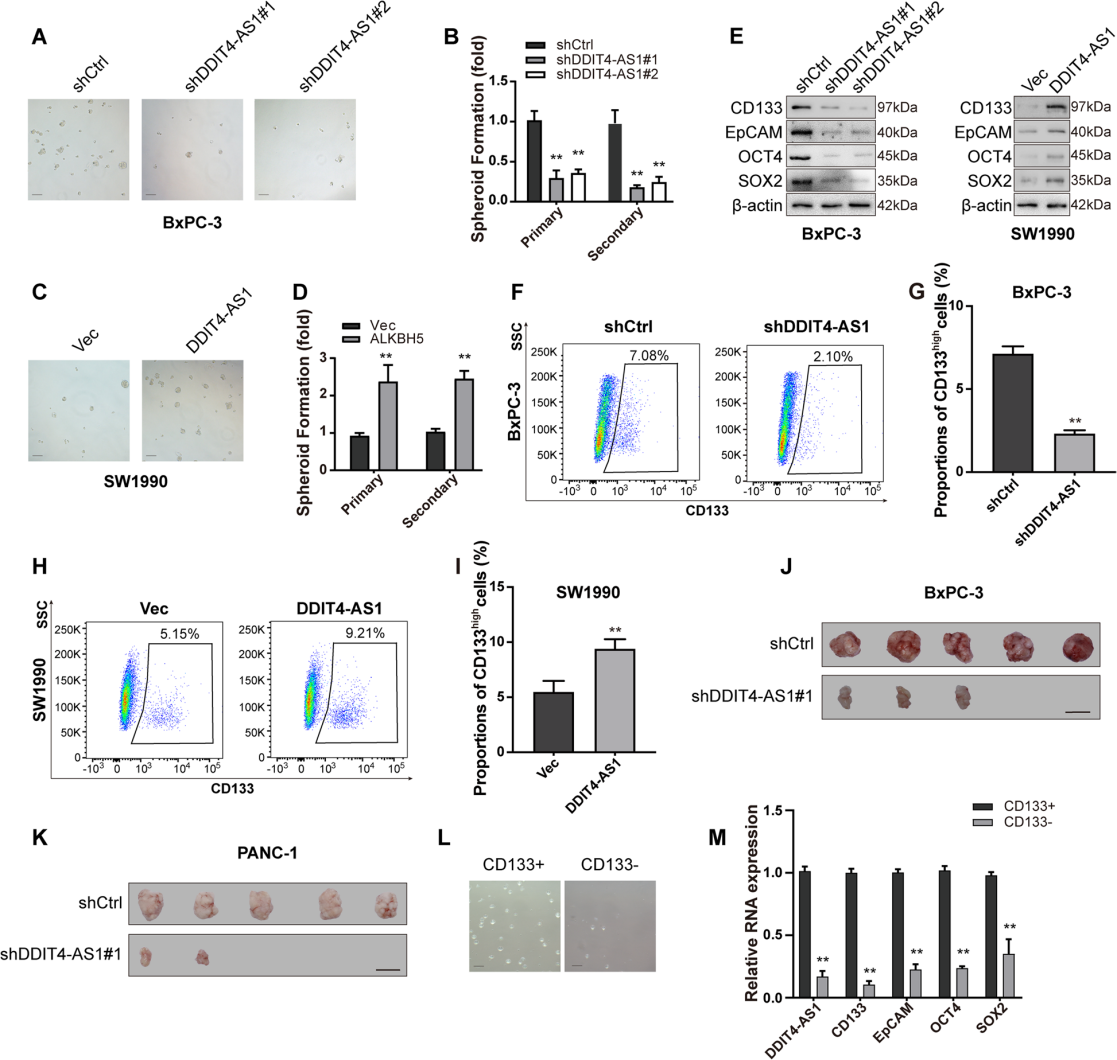
DDIT4-AS1 maintains the stemness properties of PDAC cells. **a-d** Representative images of sphere formation induced by the transfection of shDDIT4-AS1 into BxPC-3 cells or a DDIT4-AS1 overexpression plasmid into SW1990 cells. The surviving colonies were measured by calculating the fold changes (the number of tumour spheres in the DDIT4-AS1-silenced BxPC-3 cells or DDIT4-AS1-overexpressing SW1990 cells relative to that in ctrl cells). **e** The expression levels of CSC markers, including CD133, EpCAM, OCT4 and SOX2, were examined in DDIT4-AS1-silenced BxPC-3 cells or DDIT4-AS1-overexpressing SW1990 cells using Western blotting. **f-i** Flow cytometry was used to assess the percentage of CD133^high^ cells among PDAC cells with DDIT4-AS1 depletion or overexpression. **j-k** Tumour formation in nude mice injected with shDDIT4-AS1-transfected BxPC-3 and SW1990 cells. **l** Sphere formation of sorted CD133^+^ and CD133^−^ BxPC-3 cells. Only the top 2% of the most brightly stained cells or the bottom 2% of most dimly stained cells were selected as CD133^+^ or CD133^−^ populations, respectively. **m** The levels of DDIT4-AS1 and mRNAs encoding CSC markers were examined in CD133^+^ and CD133^−^ BxPC-3 cells using qRT-PCR. Data are presented as the means ± SD of three independent experiments (*, *P* < 0.05; **, *P* < 0.01)

CSCs are a small subpopulation of cells within a cancer and play a central role in cancer progression and therapy resistance, including PDAC [25]. ALKBH5 was reported to facilitate the development of several types of solid tumours and promote the self-renewal of relevant CSCs [26]. Therefore, we investigated the regulatory role of DDIT4-AS1 in PDAC stemness. Both gain- and loss-of-function studies were conducted. DDIT4-AS1 knockdown reduced the ability of PDAC cells to form primary and secondary spheroids (Fig. 3a-b, Fig. S1e-f). However, forced expression of DDIT4-AS1 increased primary and secondary spheroid formation (Fig. 3c-d). Next, the potential regulatory effect of DDIT4-AS1 on the expression of CSC markers was examined. DDIT4-AS1 silencing reduced the expression of CSC markers; conversely, DDIT4-AS1 overexpression increased the expression of CSC markers (Fig. 3e, S1g). Consistent with the results for CSC markers, the proportions of CD133^high^ PDAC cells were significantly decreased among DDIT4-AS1-depleted PDAC cells and increased among DDIT4-AS1-overexpressing PDAC cells (Fig. 3f-i, S1h-i). In vivo serial dilution experiments were applied to assess the tumour-forming ability. Mice injected with DDIT4-AS1 knockdown BxPC-3 and PANC-1 cells exhibited an appreciably reduced tumour incidence, slightly reduced tumour growth and weights compared to control mice (Fig. 3j-k, S1j-n), suggesting that the CSC frequency was significantly reduced in DDIT4-AS1-depleted PDAC cells compared to control cells.

CD133^+^ cells possess stem-like properties. We sorted CD133^+^ and CD133^−^ subsets from PDAC cells overexpressing DDIT4-AS1 using FACS. Compared to CD133^−^ cells, CD133^+^ PDAC cells expressed DDIT4-AS1 and CSC markers at higher levels and exhibited stronger spheroid formation abilities (Fig. 3l-m, S1o). GEM is used in the clinic as a standard treatment for some patients with PDAC. Consistent with the stemness characteristics, sorted CD133^+^ cells displayed a stronger ability to resist apoptosis induced by GEM treatment than CD133^−^ cells (Fig. S1p).

### DDIT4-AS1 suppresses the chemosensitivity of PDAC cells to GEM

**Fig. 4:**
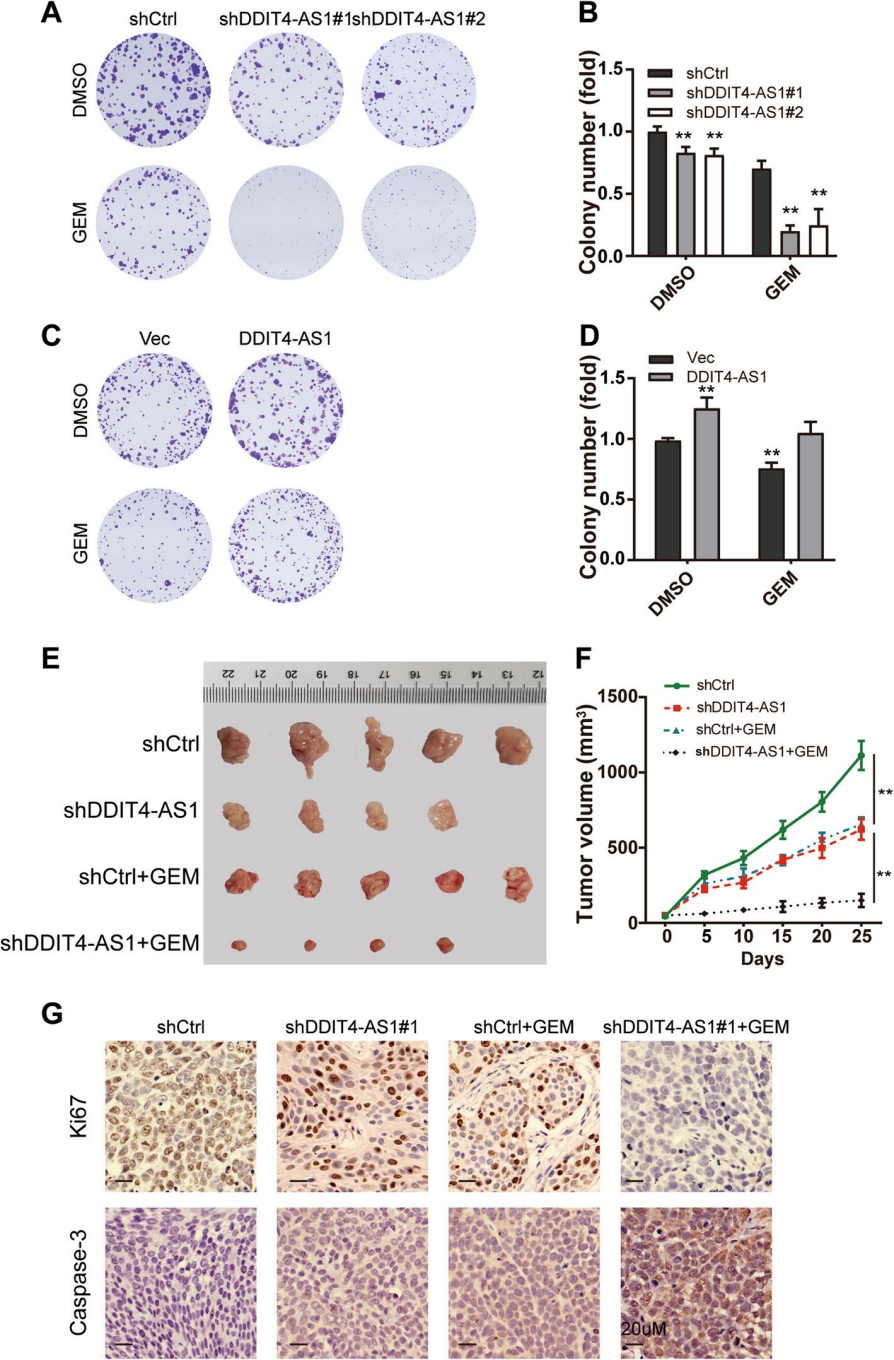
DDIT4-AS1 suppresses the chemosensitivity of PDAC cells to GEM. **a-b** Colony formation in shDDIT4-AS1-silenced PDAC cells after treatment with 10 nM GEM. **c-d** Colony formation in DDIT4-AS1 overexpression plasmid-transfected PDAC cells after treatment with 10 nM GEM. **e** Representative images of xenograft tumours in nude mice after different treatments. GEM was injected intraperitoneally twice per week for 26 days. The combination of shDDIT4-AS1 with GEM exerted stronger inhibitory effects on tumour growth. **f** The tumour growth curves of each group of mice are summarized. **g** IHC staining for Ki67 and Caspase-3 expression in different groups. Data are presented as the means ± SD of three independent experiments (*, *P* < 0.05; **, *P* < 0.01)

Then, we investigated whether DDIT4-AS1 manipulates the chemosensitivity of PDAC cells. Ectopic suppression of DDIT4-AS1 sensitized PDAC cells to GEM, as reflected by the reduced colony formation ability (Fig. 4a-b, S2a-b). In contrast, overexpression of DDIT4-AS1 compromised GEM-induced suppression of colony formation (Fig. 4c-d). Except for GEM, we also tested the chemosensitivity of cancer cells to paclitaxel, The combination of shDDIT4-AS1 with paclitaxel had similar inhibitory effects on tumor growth, but weaker than the combination effects of shDDIT4-AS1 with GEM (Fig. S2c-d).

We next evaluated the role of DDIT4-AS1 in regulating GEM sensitivity in vivo by subcutaneously injecting equal amounts of control or knockdown PDAC cells (2 × 10^6^) into mouse flanks. After one week, the nude mice were treated with 60 mg/kg GEM twice a week. Tumour growth in each group was monitored for 26 days. The DDIT4-AS1 knockdown group treated with GEM exhibited substantial reductions in tumour weight and tumour growth (Fig. 4e-f). Moreover, the combination of DDIT4-AS1 knockdown and GEM treatment resulted in weak staining for Ki67 and strong staining for Caspase-3 (Fig. 4g, S2e). Overall, these data suggested that the tumours derived from PDAC cells with DDIT4-AS1 knockdown were more sensitive to GEM than those derived from vector-transfected PDAC cells.

### DDIT4-AS1 regulates stemness and chemosensitivity by activating the mTOR pathway

**Fig. 5:**
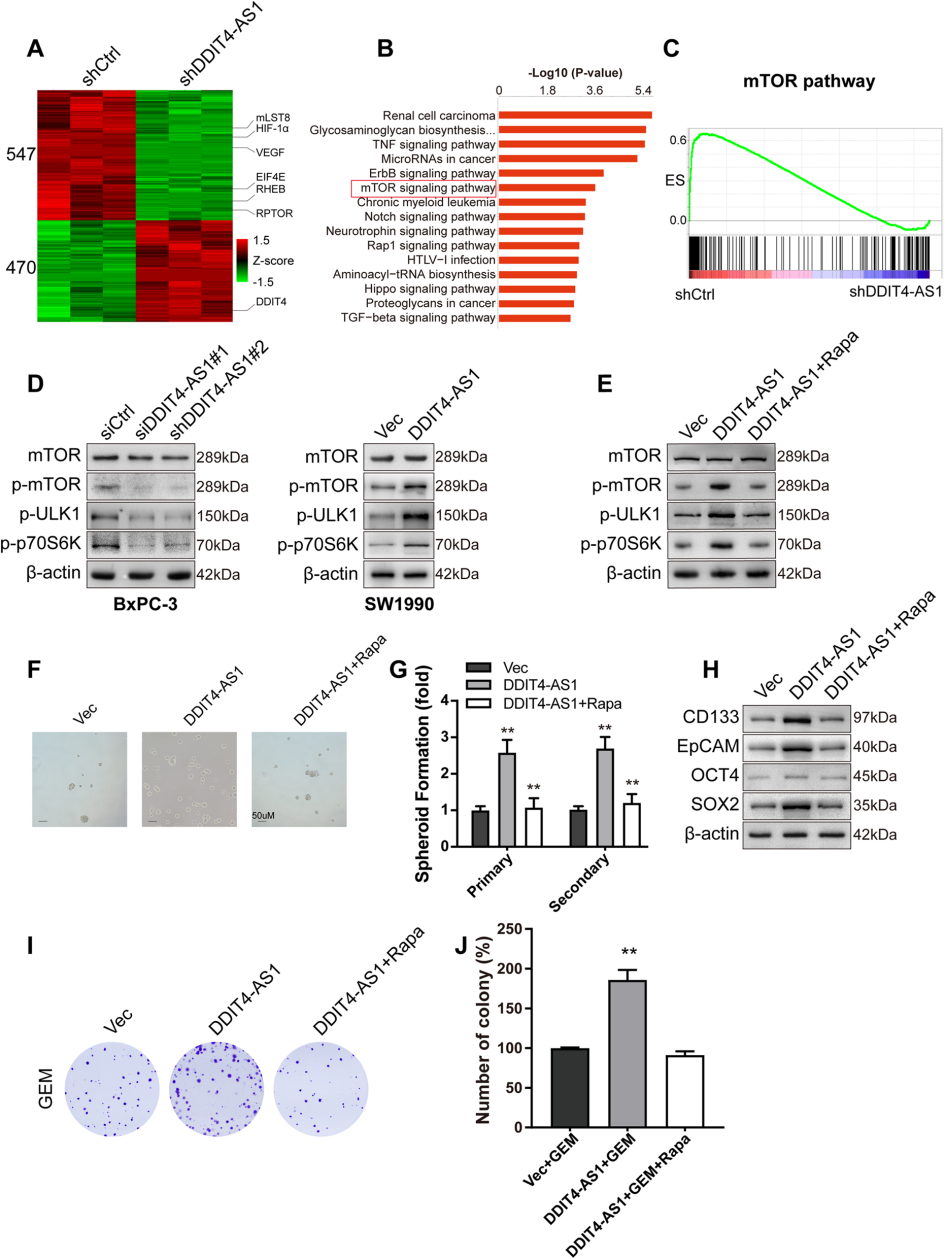
DDIT4-AS1 regulates stemness and chemosensitivity by activating the mTOR pathway. **a** Heatmap of the differentially expressed genes after DDIT4-AS1 knockdown in BxPC-3 cells. **b** KEGG analysis of the differentially expressed genes after DDIT4-AS1 knockdown. **c** GSEA showed that differentially expressed genes identified following DDIT4-AS1 knockdown were enriched in the mTOR pathway. **d** WB analysis of mTOR pathway-related proteins in BxPC-3 cells with DDIT4-AS1 depletion and SW1990 cells with DDIT4-AS1 overexpression. **e** WB analysis of mTOR pathway-related proteins in DDIT4-AS1-overexpressing cells with or without rapamycin treatment. **f-g** Primary sphere formation was assessed in DDIT4-AS1-depleted BxPC-3 cells with or without rapamycin treatment. **h** The expression of CSC markers was examined using WB in DDIT4-AS1-depleted BxPC-3 cells with or without rapamycin treatment. **i-j** Colony formation was examined in shDDIT4-AS1-silenced BxPC-3 cells with or without rapamycin treatment after treatment with GEM. Data are presented as the means ± SD of three independent experiments (*, *P* < 0.05; **, *P* < 0.01)

We explored the potential mechanisms by which DDIT4-AS1 regulates stemness and chemosensitivity by performing RNA sequencing (RNA-seq) to identify the differentially expressed genes upon DDIT4-AS1 knockdown, and 547 upregulated genes and 470 downregulated genes were identified (|LogFC|≥ 1, *P* < 0.05, Fig. 5a). KEGG enrichment revealed that the significantly enriched pathways include TNF, ErbB and mTOR pathway (Fig. 5b). We supplemented Etanercept and Dacomitinib to DDIT4-AS1-overexpression-BxPC-3 cells and found that the TNF or ErbB signaling pathway did not have obvious effects on the stemness of pancreatic cancer cells (Fig. S2f-i). Multiple studies have reported that the mTOR pathway may contribute to cancer stemness maintenance [27, 28], chemoresistance [29–31] and tumour development [32]. The GSEA results showed that the differentially expressed gene sets were significantly related to the mTOR pathway (Fig. 5c). Next, we evaluated whether mTOR pathway is involved in stemness regulation mediated by DDIT4-AS1. The levels of proteins in the mTOR signalling pathway were examined using western blotting, and the results showed that DDIT4-AS1 knockdown inhibited mTOR pathway activity, as reflected by the decreased phosphorylation of mTOR, ULK1 and p70S6K. Meanwhile, DDIT4-AS1 overexpression activated the mTOR pathway (Fig. 5d). Then, we assessed whether the mTOR pathway was required for the DDIT4-AS1-mediated regulation of stemness and chemosensitivity. PDAC cells overexpressing DDIT4-AS1 were exposed to rapamycin to inhibit mTOR pathway activity (Fig. 5e). As expected, the treatment of cells with rapamycin restored the effects of DDIT4-AS1 overexpression on stemness marker expression, spheroid formation ability and chemosensitivity to GEM (Fig. 5f-j). These results indicate that DDIT4-AS1 promotes stemness and inhibits chemosensitivity by activating the mTOR pathway.

### DDIT4-AS1 recruits UPF1 to destabilize DDIT4 and activate the mTOR pathway

**Fig. 6:**
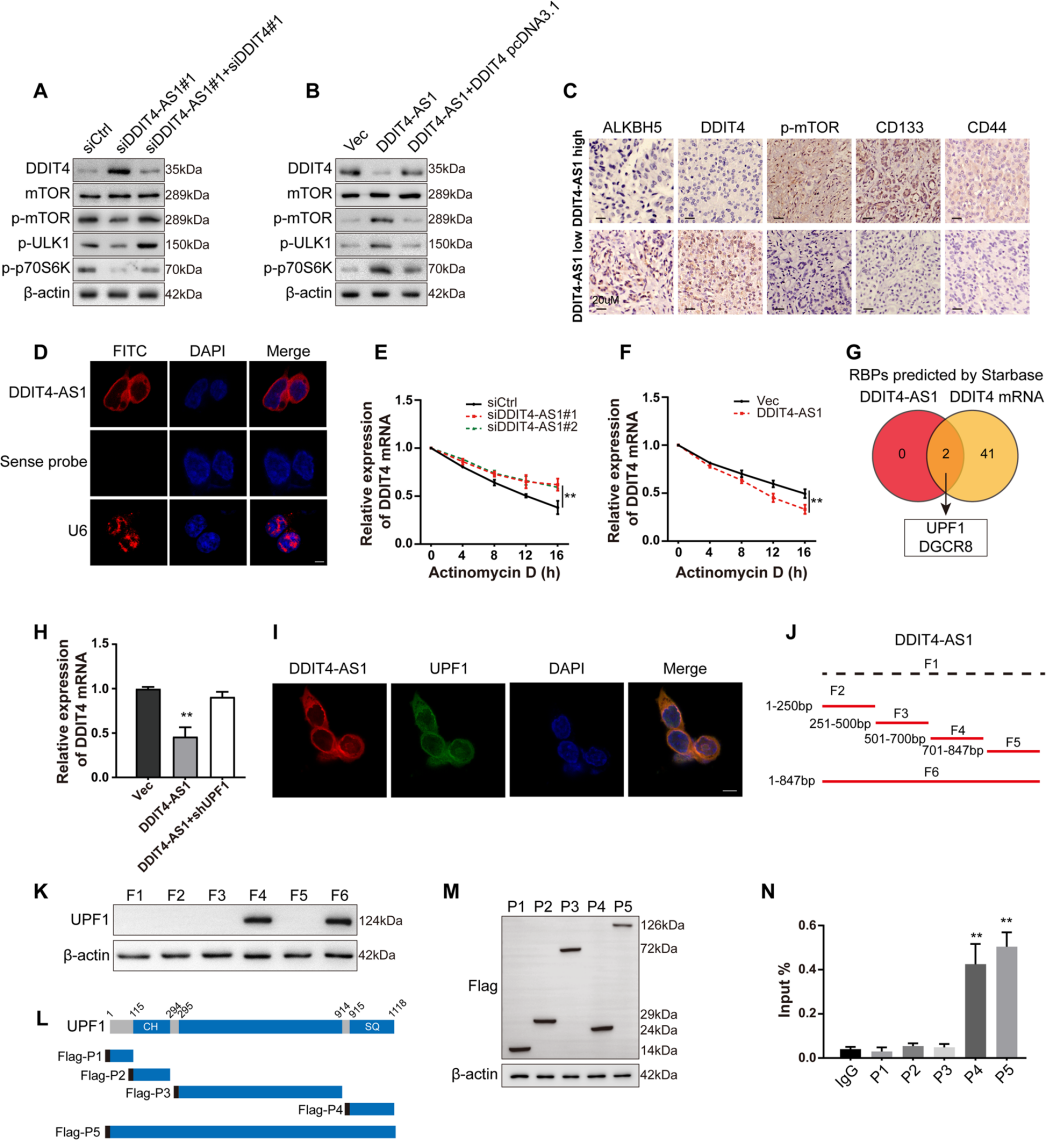
DDIT4-AS1 recruits UPF1 to destabilize DDIT4 and activate the mTOR pathway. **a** WB analysis of mTOR pathway-related proteins in BxPC-3 cells cotransfected with the DDIT4-AS1 siRNA and/or DDIT4 siRNA. **b** WB analysis of mTOR pathway-related proteins in SW1990 cells cotransfected with the exogenous DDIT4-AS1 plasmid and/or DDIT4 plasmid. **c** IHC staining showing the expression of ALKBH5, DDIT4, p-mTOR, CD133 and CD44 in PDAC tissues with higher or lower levels of DDIT4-AS1 formed by shDDIT4-AS1 or control cells. **d** RNA-FISH assay showing the cytoplasmic localization of DDIT4-AS1 in BxPC-3 cells incubated with DDIT4-AS1 probe (red) and nuclear staining with DAPI (blue). U6 was applied as a positive control (red). **e–f** The decay rate of the DDIT4 mRNA after treatment with 2.5 μM actinomycin D for the indicated times following DDIT4-AS1 knockdown in BxPC-3 cells or DDIT4-AS1 overexpression in SW1990 cells. **g** Venn diagram showing the overlapping proteins that might interact with DDIT4-AS1 and the DDIT4 mRNA, which were predicted using starBase. **h** The level of DDIT4 mRNA was examined in DDIT4-AS1-overexpressing SW1990 cells with or without UPF1 depletion using qRT‒PCR. **i** The colocalization analysis was performed with specific probes against DDIT4-AS1 (red) and a specific antibody against UPF1 (green). Nuclei were stained with DAPI. **j** Domain mapping of full-length (F6), truncated DDIT4-AS1 (F2-F5) and the antisense nucleotides of DDIT4-AS1 (F1). **k** WB showing UPF1 levels in samples pulled down by F1-F6. **l** Domain mapping of Flag-labelled full-length UPF1 (P5) or truncated UPF1 (P1-P4). **m** WB analysis of Flag-tagged UPF1 constructs in BxPC-3 cells transfected with each construct (P1: 1–115 aa, P2: 116–294 aa, P3: 295–914 aa, P4: 915–1118 aa, and P5: 1–1118 aa). **n** qRT-PCR was used to determine the expression of DDIT4-AS1 immunoprecipitated by the anti-Flag antibody targeting recombinant proteins. Data are presented as the means ± SD of three independent experiments (*, *P* < 0.05; **, *P* < 0.01)

The cognate sense transcript of DDIT4-AS1 is DDIT4, which has been identified as a negative regulator of the mTOR signalling pathway [33]. Interestingly, when we referred to the gene set of the mTOR signalling pathway, DDIT4 was significantly upregulated upon DDIT4-AS1 knockdown. The WB results indicated that DDIT4 silencing reactivated the mTOR pathway that was suppressed by DDIT4-AS1 depletion (Fig. 6a), whereas DDIT4 overexpression abolished the activation of the mTOR pathway caused by DDIT4-AS1 overexpression (Fig. 6b). Compared to the patients with lower levels of DDIT4-AS1, patients with high levels of DDIT4-AS1 showed higher levels of p-mTOR, CD133 and CD44 IHC staining and lower levels of ALKBH5 and DDIT4 (Fig. 6c, S2j). The IHC staining of subcutaneous tumour tissues revealed decreased p-mTOR and increased DDIT4 levels in the sh-DDIT4-AS1 group compared with the shCtrl group (Fig. S2k-l). Based on these results, DDIT4 mediates the regulation of the mTOR pathway by DDIT4-AS1. There have been studies demonstrated that the effects of DDIT4 on mTOR was dependent on TSC complex, especially TSC2 [34, 35]. In order to verify above mechanism, TSC2 was knockdown or overexpression with siRNA or overexpression plasmid. The effects of DDIT4-AS1 and DDIT4 on mTOR were abrogated in cells transfected with TSC2 siRNAs or overexpression plasmid (Fig. S2m-n), suggesting the function of DDIT4 on mTOR required TSC complex.

We next examined the mechanism by which DDIT4-AS1 regulates DDIT4. FISH staining showed that DDIT4-AS1 was located in the cytoplasm of PDAC cells (Fig. 6d). DDIT4-AS1 knockdown resulted in an increase in the half-life of the DDIT4 mRNA (Fig. 6e), whereas DDIT4-AS1 overexpression led to a decreased half-life of the DDIT4 mRNA (Fig. 6f), suggesting that DDIT4-AS1 destabilizes the DDIT4 mRNA. Then, we performed an RNA pull-down assay using a biotinylated DDIT4-AS1 oligonucleotide, and endogenous DDIT4 mRNA did not coprecipitate with DDIT4-AS1; therefore, we hypothesized that some protein(s) may mediate the regulatory effect of DDIT4-AS1 on the DDIT4 mRNA. We used the starBase database to predict the proteins that interact with DDIT4-AS1 and DDIT4, and UPF1 and DGCR8 were identified as potential interactors (Fig. 6g). We selected UPF1, which may participate in mRNA degradation, for further validation. Knockdown of UPF1 in DDIT4-AS1-overexpressing cells rescued the decreased expression of the DDIT4 mRNA (Fig. 6h). In contrast, overexpression of UPF1 in DDIT4-silenced cells exerted the opposite effects (Fig. S2o), suggesting that UPF1 facilitated the DDIT4-AS1-mediated degradation of DDIT4.

We evaluated the interaction between DDIT4-AS1 and UPF1 by performing IF colocalization assays and showed that DDIT4-AS1 and UPF1 colocalized in the cytoplasm of PDAC cells (Fig. 6i). RIP assays with an UPF1 antibody showed that UPF1 bound specifically to DDIT4-AS1 (Fig. S2p). Then, we detected the binding sites for DDIT4-AS1 with UPF1. We constructed one biotinylated full-length anti-sense DDIT4-AS1 probe (F1), four biotinylated fragments of DDIT4-AS1 probes (F2-F5) and one full-length sense DDIT4-AS1 probe (F6) to pull down UPF1 in PDAC cell lysates (Fig. 6j). The fragment consisting of bases 701 to 847 of DDIT4-AS1 was crucial for the interaction with UPF1, whereas other fragments did not interact with UPF1 (Fig. 6k). As previously reported, UPF1 contains two major domains: the SQ domain near the C-terminus and the CH domain near the N-terminus (Fig. 6l). We identified the binding region in UPF1 by cloning a series of Flag-tagged UPF1 constructs (P1: 1–115 aa, P2: 116–294 aa, P3: 295–914 aa, P4: 915–1118 aa, and P5: 1–1118 aa) (Fig. 6m). RIP assays were performed with an anti-Flag antibody and revealed that both P4 and P5 interacted with DDIT4-AS1 (Fig. 6n), suggesting that the SQ domain of UPF1 is required for the interaction with DDIT4-AS1.

Then, we further verified whether the DDIT4 mRNA bound to UPF1. We performed RIP in PDAC cells and found that UPF1 bound to the DDIT4 mRNA (Fig. S3a). We determined the region in which DDIT4 participates in the interaction by constructing a Flag-tagged expression vector including the DDIT4 coding sequence with the 3'UTR (DDIT4 CDS-3'UTR) or the CDS alone (DDIT4 CDS) and cotransfected PDAC cells with the UPF1 siRNA or overexpression plasmid. UPF1 knockdown or overexpression negatively regulated the expression of DDIT4 in PDAC cells transfected with the DDIT4 CDS-3'UTR. However, its expression was not altered in cells transfected with the DDIT4 CDS alone (Fig. S3b-i), suggesting that the 3'UTR is indispensable for the UPF1-mediated regulation of DDIT4.

### DDIT4-AS1 prevents the binding of SMG5 and PP2A to UPF1 and inhibits its phosphorylation

**Fig. 7:**
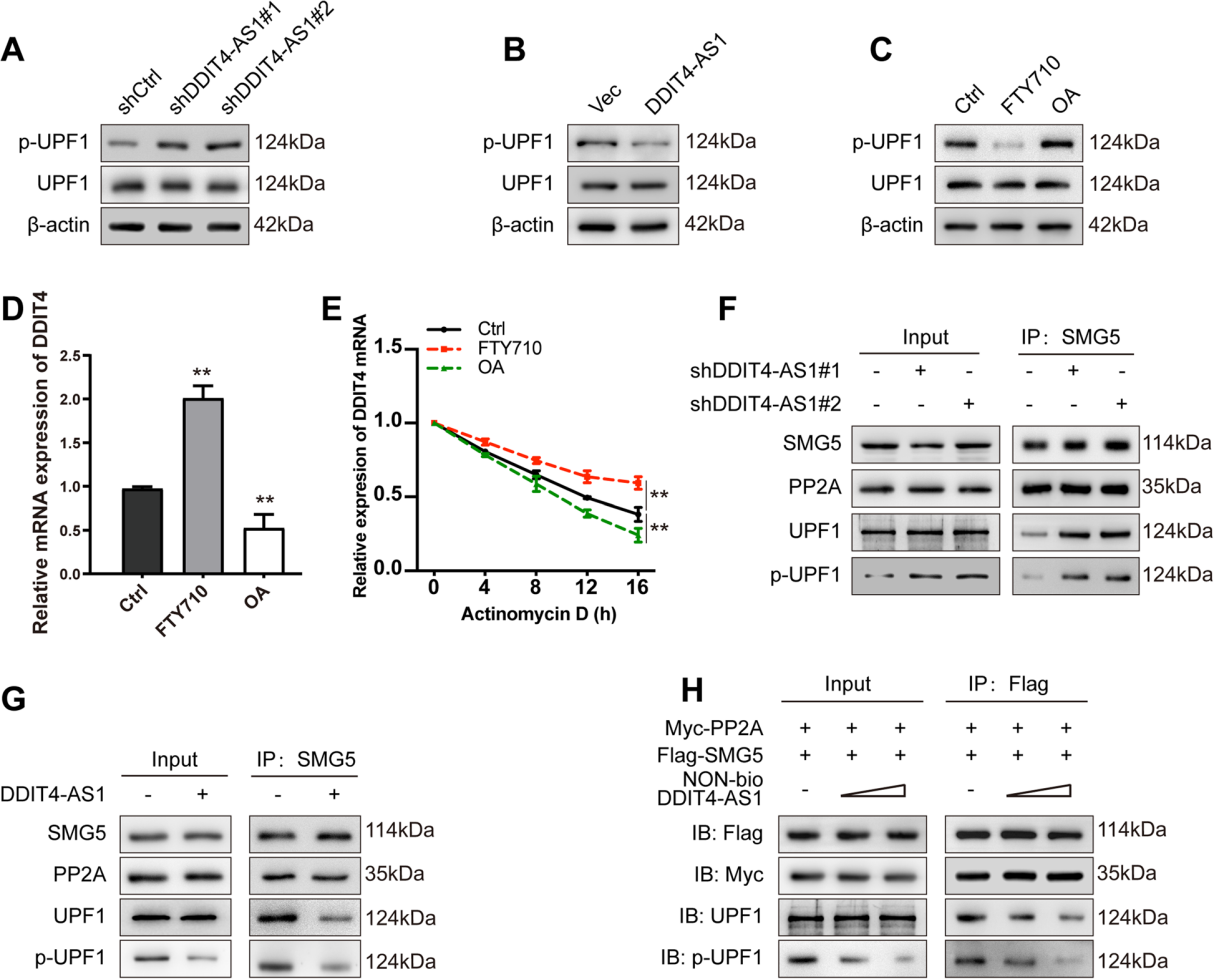
DDIT4-AS1 prevents the binding of SMG5 and PP2A to UPF1 and inhibits UPF1 phosphorylation. **a-b** WB analysis of levels of the phosphorylated UPF1 and total UPF1 proteins in PDAC cells transfected with the DDIT4-AS1 siRNA or DDIT4-AS1 overexpression plasmid. **c** WB analysis of levels of the phosphorylated UPF1 and total UPF1 proteins in BxPC-3 cells treated with FTY710 or OA. **d** qRT-PCR analysis of the DDIT4 mRNA in BxPC-3 cells treated with FTY710 or OA. **e** The decay rate of the DDIT4 mRNA after treatment with 2.5 μM actinomycin D for the indicated times following treated with FTY710 or OA in BxPC-3 cells. **f** BxPC-3 cells were transfected with or without the DDIT4-AS1 siRNA, lysates were subjected to IP using anti-SMG5 antibodies, and coprecipitates were subjected to WB detection. **g** SW1990 cells were transfected with or without the DDIT4-AS1 overexpression plasmid, lysates were subjected to IP using anti-SMG5 antibodies, and coprecipitates were subjected to WB detection. **h** 293 T cells were transfected with myc-tagged PP2A and flag-tagged SMG5 and treated with or without nonbiotinylated DDIT4-AS1. Lysates were subjected to IP using anti-Flag antibodies, and coprecipitates were analysed using WB. Data are presented as the means ± SD of three independent experiments (*, *P* < 0.05; **, *P* < 0.01)

Phosphorylation of UPF1 is required for its functions and plays an important role in regulating RNA stability. Notably, the depletion of DDIT4-AS1 induced the accumulation of phospho-UPF1 without affecting the total amount of UPF1 (Fig. 7a). However, DDIT4-AS1 overexpression exerted the opposite effects (Fig. 7b). According to a previous study, the SMG5 interaction with PP2A is required for the dephosphorylation of UPF1 in an evolutionarily conserved manner [36]. We treated PDAC cells with okadaic acid (OA, a potent inhibitor of PP2A) or FTY710 (an activator of PP2A). OA treatment resulted in an increase in p-UPF1 levels, coincident with a decrease in DDIT4 mRNA expression and stability (Fig. 7c-e); in contrast, FTY710 produced the opposite changes in p-UPF1 level, DDIT4 mRNA expression and stability (Fig. 7c-e). We further evaluated the underlying mechanism by performing immunoprecipitation in cells with DDIT4-AS1 knockdown and overexpression, and the results showed that SMG5 and PP2A interacted with UPF1 and phospho-UPF1. This interaction was attenuated by DDIT4-AS1 knockdown (Fig. 7f) and enhanced by exotic expression of DDIT4-AS1 (Fig. 7g). Then, we transiently co-expressed epitope-tagged PP2A and SMG5 in 293 T cells treated with nonbiotinylated DDIT4-AS1, finding that nonbiotinylated DDIT4-AS1 suppresses the interaction of SMG5 and PP2A with UPF1 and phospho-UPF1 in a dose-dependent manner (Fig. 7h).

### The combination of DDIT4-AS1 knockdown and GEM exerts synergistic effects on PDX models

**Fig. 8:**
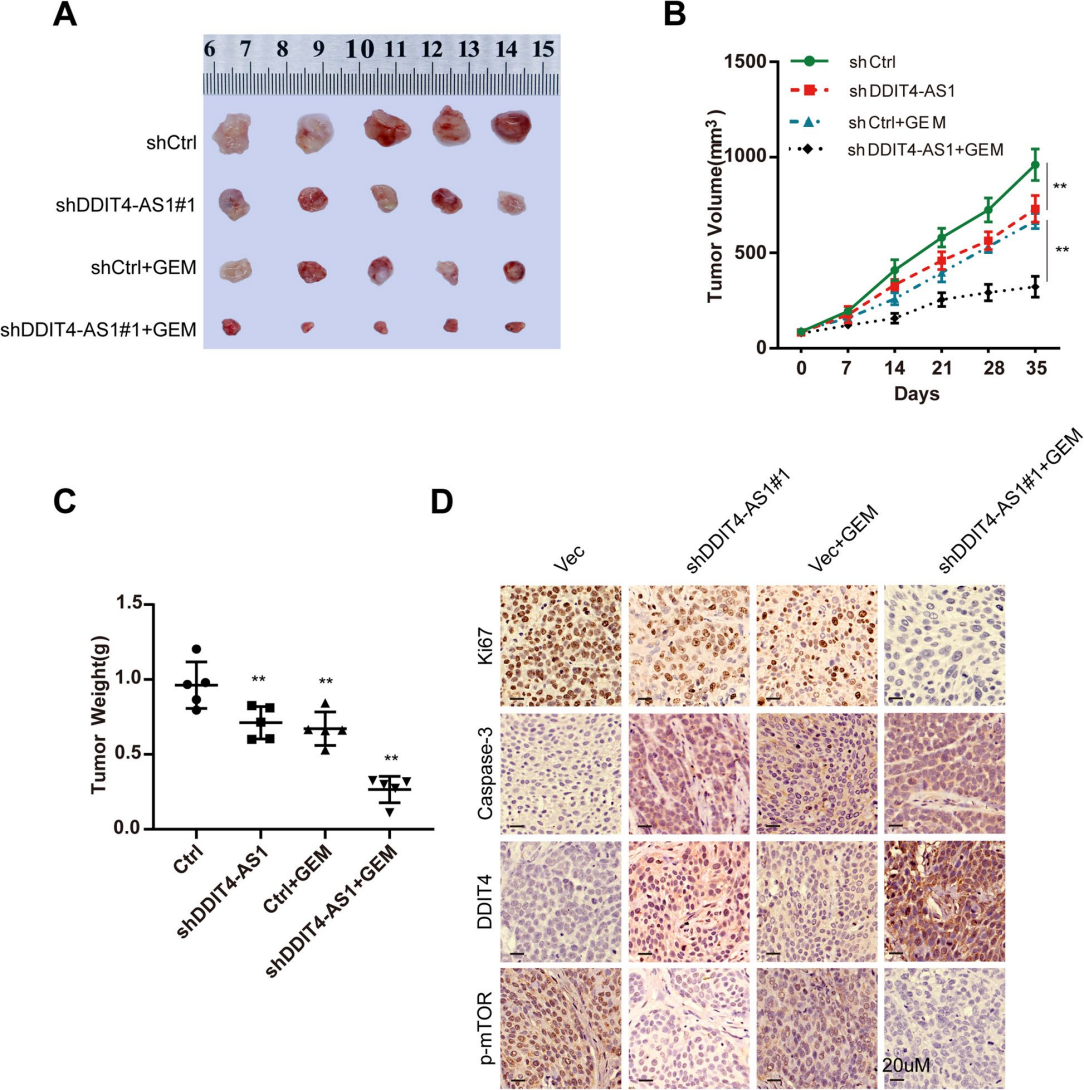
The combination of DDIT4-AS1 knockdown and GEM exerts synergistic effects on PDX models. **a-b** Representative images and growth curves of PDX xenografts from mice after different treatments. shCtrl or shDDIT4-AS1 was injected intratumourally once per week for 5 weeks. GEM was injected intraperitoneally twice per week for 5 weeks. **c** The tumour weight of PDX xenografts from mice that received different treatments for 5 weeks. **d** The levels of Ki67, Caspase-3, DDIT4 and p-mTOR were assessed in different groups using IHC. Data are presented as the means ± SD of three independent experiments (*, *P* < 0.05; **, *P* < 0.01)

We conducted an in vivo evaluation of the efficacy of the combination of DDIT4-AS1 knockdown and GEM therapy in PDX models, which were successfully established in our lab. The nude mice were divided into 4 groups (*n* = 5 per group) and received an intratumour injection of an empty vector (Vec) or lentiviral-shDDIT4-AS1 (shDDIT4-AS1); meanwhile, 2 of 4 groups also received an intraperitoneal injection of GEM. The combination of shDDIT4-AS1 with GEM exerted a stronger inhibitory effect on tumour growth and weight than any individual treatment (Fig. 8a-c). IHC staining for DDIT4 and p-mTOR showed that DDIT4-AS1 silencing increased DDIT4 expression and reduced p-mTOR expression (Fig. 8d, S3j). Moreover, the combination of shDDIT4-AS1 with GEM reduced proliferation and increased apoptosis compared with the other groups, as determined using Ki67 and Caspase-3 staining (Fig. 8d, S3j). Taken together, virus-mediated DDIT4-AS1 silencing increased the chemosensitivity of PDAC cells to GEM through the DDIT4-mediated mTOR pathway.

## Discussion

**Fig. 9:**
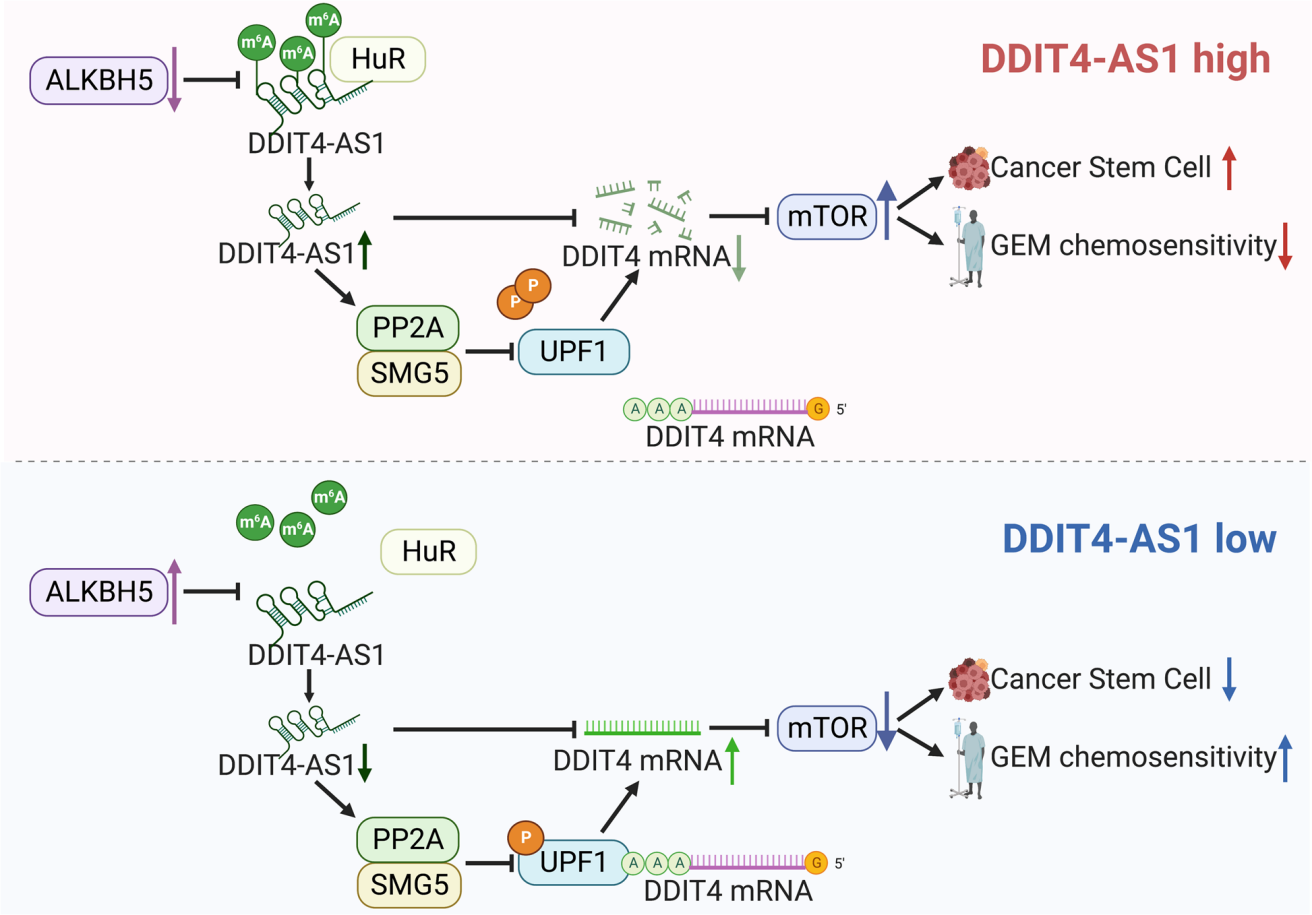
The schematic figure of the mechanisms. (Created with BioRender.com.)

The m^6^A modification is one of the most common epigenetic methylation modifications and is involved in almost all stages of RNA metabolism, including RNA splicing, export, stability, and translation [37]. In addition to mRNAs, noncoding RNAs, such as lncRNAs, are also substrates of m^6^A modification. In the present study, we found that ALKBH5 mediated m^6^A demethylation of DDIT4-AS1 and that HuR stabilized DDIT4-AS1 by binding to m^6^A sites. HuR is recognized as an m^6^A reader, and a previous study reported that HuR was recruited to m^6^A-containing RNA and contributed to RNA stabilization [38], consistent with our study. Our study also revealed that DDIT4-AS1 was upregulated in PDAC tissues and that increased DDIT4-AS1 levels predicted poor clinical outcomes. Therefore, DDIT4-AS1 may serve as a potential diagnostic and prognostic biomarker (Fig. 9).

DDIT4-AS1 is functionally related to controlling the stemness properties and chemosensitivity of PDAC cells. Overexpression of DDIT4-AS1 enhances stemness and suppresses chemosensitivity to GEM. We explored the underlying mechanism by performing RNA-seq to examine the differentially expressed genes, and the mTOR pathway was identified as an enriched pathway. mTOR is a 289 kDa serine/threonine kinase belonging to the PI3K-related kinase family [39]. mTOR encompasses two functionally distinct protein complexes: mTORC1 and mTORC2 [40]. The serine/threonine kinases p70S6K1 [41] and ULK1 [42] are the most well-known downstream targets of mTORC1. mTOR is frequently dysregulated in various human cancers and plays a crucial role in multiple biological processes, including stemness and chemosensitivity regulation [43–45]. For example, miR-135b silencing inactivates the AKT/mTOR pathway and ultimately results in the inhibition of self-renewal and tumour growth of pancreatic cancer stem cells [46]. PRKAR1B-AS2 promotes tumour growth and confers chemoresistance by modulating the PI3K/AKT/mTOR pathway in ovarian cancer [47]. In the present study, DDIT4-AS1 knockdown decreased p-mTOR, p-p70S6K and p-ULK1 levels to inhibit the mTOR pathway. Rapamycin restored the effects of DDIT4-AS1 overexpression on stemness and chemosensitivity to GEM in pancreatic cancer. Thus, the effect of DDIT4-AS1 depends on the mTOR signalling pathway.

Antisense lncRNAs were identified to have a close relationship with homologous sense genes in a natural physiological state [48]. Some antisense lncRNAs regulate sense mRNAs. KRT7-AS, which functions as an oncogene in gastric cancer, was speculated to form a complementary complex with its sense KRT7 mRNA and affect KRT7 mRNA stability [49]. SATB2-AS1 is the cognate antisense transcript of SATB2, and SATB2-AS1 activates SATB2 in cis by recruiting WDR5 and GADD45A to the SATB2 promoter region and inhibits the progression of colorectal cancer [50]. As DDIT4 is the sense mRNA of DDIT4-AS1, we evaluated whether DDIT4 was involved in the regulation of mTOR activation by DDIT4-AS1. DDIT4 silencing reactivated the mTOR pathway that was suppressed by DDIT4-AS1 depletion, whereas DDIT4 overexpression abolished the activation of the mTOR pathway caused by DDIT4-AS1 overexpression, indicating that DDIT4 mediates the regulation of the mTOR pathway by DDIT4-AS1.

In terms of the relationship between DDIT4-AS1 and DDIT4, the RNA pull-down results suggested that DDIT4-AS1 did not directly interact with DDIT4. Therefore, we conducted a bioinformatics analysis to determine whether some regulatory proteins were involved in this process, and UPF1 was identified as a possible molecule. UPF1 is an RNA helicase belonging to helicase superfamily 1 that has two major domains: an SQ domain near the C-terminus and a CH domain near the N-terminus. UPF1 is a well-known key factor involved in nonsense-mediated mRNA decay (NMD), a conserved mRNA surveillance pathway that degrades mRNA containing a premature termination codon [51]. For example, UPF1 directly interacts with the STAU1 mRNA to promote its degradation [52]. In our study, we confirmed that knockdown of UPF1 rescued the decreased expression of the DDIT4 mRNA induced by DDIT4-AS1 overexpression and vice versa. The interaction of UPF1 with DDIT4-AS1 and DDIT4 was validated by RIP and RNA pulldown, indicating that UPF1 mediated the regulatory effect of DDIT4-AS1 on the DDIT4 mRNA.

Previous studies revealed that UPF1 phosphorylation plays an important role in promoting its cellular activities and facilitates the binding of other factors involved in NMD and other processes to it [53]. Interestingly, DDIT4-AS1 suppressed the phosphorylation of UPF1 without affecting the amount of UPF1 protein in the present study. However, the underlying mechanism is unknown. SMG1, SMG5, SMG6 and SMG7 are essential NMD factors that have been implicated in the regulation of UPF1 phosphorylation [54]. Among these NMD factors, SMG5 directly recruits PP2A phosphatases via the C-terminal PIN domain and results in dephosphorylation of UPF1 [53]. In our study, we confirmed that SMG5 and PP2A coimmunoprecipitated with UPF1, and treating PDAC cells with a potent inhibitor or activator of PP2A results in an increase or decrease in p-UPF1 levels, respectively, consistent with previous reports. Further observations revealed that this interaction between SMG5, PP2A and UPF1 was attenuated by DDIT4-AS1 knockdown and enhanced by ectopic expression of DDIT4-AS1, which supports the hypothesis that DDIT4-AS1 dissociates SMG5 and PP2A from UPF1 and enhances NMD by increasing UPF1 phosphorylation. It was also reported that SMG5 can bind to SMG7 to form a complex that preferentially binds to phosphorylated UPF1[36], SMG6 had also been proposed to compete with the SMG5:SMG7 complex for binding to phospho-UPF1[55]. Whether SMG7 and SMG6 are involved in regulation of UPF1 phosphorylation by DDIT4-AS1 is unknown and remains to be clarified in our future studies.

## Conclusions

In conclusion, DDIT4-AS1 is amplified, overexpressed, and stabilized by ALKBH5 and HuR in PDAC cells. DDIT4-AS1, which functions as an oncogene, significantly promotes PDAC stemness and suppresses chemosensitivity to GEM. DDIT4-AS1 physically interacts with UPF1 and covers the binding sites for SMG5 and PP2A to promote UPF1 phosphorylation, the degradation of the DDIT4 mRNA and activation of the mTOR pathway. Treatment with the DDIT4-AS1 shRNA combined with GEM significantly increased the antitumour activity of PDAC in a PDX-based model. The discovery of DDIT4-AS1 provides insights into PDAC carcinogenesis and may facilitate the development of precise approaches for cancer screening and treatment.

## Supplementary Information

**Additional file 1: Figure S1. a**The RNA levels of DDIT4-AS1 in 9 pairedhuman PDAC tissues and normal adjacent tissues. **b** The relative RNAlevels of DDIT4-AS1 in BxPC-3cells cotransfected with the ALKBH5overexpression plasmid and DDIT4-AS1-WT or DDIT4-AS1-Mut. **c**WB and RT-PCRanalyses of IGF2BP1 and DDIT4-AS1 levels in BxPC-3 cells transfectedwith IGF2BP1 siRNAs. **d** qRT-PCR was used to determine the level of DDIT4-AS1 immunoprecipitatedby the anti-HuR antibody. **e-f** Representative images of sphere formation induced by thetransfection of shDDIT4-AS1 into PANC-1cells. The surviving colonies were measured by calculating the fold changes. **g** The expressionlevels of CSC markers were examined in DDIT4-AS1-silenced PANC-1 cells using Western blotting. **h-i** Flow cytometrywas performed to assess the percentage of CD133^high^ cells among PANC-1 cells withDDIT4-AS1 depletion. **j**BxPC-3cells with or without DDIT4-AS1 depletion were injected into the subcutaneoustissues of nude mice at a density of 5 × 10^4^, 5 × 10^3^, 5× 10^2^ or 5 × 10^1^ cells per mouse. The number of mice thathad developed tumours was counted. The frequency of CSCswas calculated using ELDA software. **k-l **Growth curves and tumour weight of nudemice after BxPC-3 cells with or without DDIT4-AS1 depletion wereinjected. **m-n **Growth curves and tumour weight of nudemice after PANC-1 cells with or without DDIT4-AS1 depletion wereinjected. **o **Sphere formation of sorted CD133^+^ and CD133^-^BxPC-3 cells. The spheroid formation were measured bycalculating the fold changes (the number of tumour spheres in theDDIT4-AS1-silenced PANC-1 cells relative tothat in ctrl cells). **p**Sorted CD133^+^ cells and CD133^-^ cells were treated withGEM. The apoptotic rate was detected using flow cytometry. Data are presented asthe means ± SD of three independent experiments (*, *P* < 0.05; **,*P*<0.01).

**Additional file 2: Figure S2. a-b**Colony formation by shDDIT4-AS1-silenced PANC-1cells after treatment with 10nM GEM. **c-d** Colony formation byshDDIT4-AS1-silenced BxPC-3 cells aftertreatment with 5uM Paclitaxel. **e **The nudemice were injected of BxPC-3 cells with orwithout DDIT4-AS1 depletion and treated with 60 mg/kg GEM twice a week. TheKi67 and Caspase-3 expression were evaluated by IHC staining. **f-g**Representative images of sphere formation in DDIT4-AS1-depleted BxPC-3 cells with or without etanercept treatment. Thespheroid formation was measured by calculating the fold changes.** h-i**Representative images of sphere formation in DDIT4-AS1-depleted BxPC-3 cells with or without dacomitinib treatment.The spheroid formation was measured by calculating the fold changes. **j **IHCstaining showing the expression of ALKBH5, DDIT4, p-mTOR, CD133 and CD44 inPDAC tissues with higher or lower levels of DDIT4-AS1 formedby shDDIT4-AS1 or control cells. **k-l**The levels of Ki67, DDIT4 and p-mTOR were assessed in the shCtrl and shDDIT4-AS1#1 groups using IHC. **m-n **WB analysis of TSC2, DDIT4 andmTOR pathway-related proteins inDDIT4-AS1-depleted BxPC-3 cells transfectedwith TSC2 siRNA and DDIT4-depleted SW1990 cells transfected with TSC2 overexpressionplasmid. **o**Thelevel of DDIT4 mRNA was examined in DDIT4-AS1-knockdown BxPC-3 cells with or without UPF1 overexpression using qRT-PCR.**p **qRT-PCR was used to determine the level of DDIT4-AS1immunoprecipitated by the anti-UPF1 antibody. Data are presented as the means ±SD of three independent experiments (*, *P* < 0.05; **, *P*<0.01).

**Additional file 3: Figure S3. a**qRT-PCR was performed to determine the level of the DDIT4 mRNAimmunoprecipitated by the anti-UPF1 antibody. **b-c** qRT-PCR analysis ofFlag-DDIT4 levels in BxPC-3cells expressing Flag-DDIT4 with the DDIT4 CDS-3'UTR or DDIT4 CDS and treatedwith control or UPF1 siRNAs. The sequence of the forwardprimer corresponds to the 3×Flag vector. **d-e** WB analysis of Flag-DDIT4levels in BxPC-3 cells expressing Flag-DDIT4 with the DDIT4 CDS-3'UTRor DDIT4 CDS and treated with control or UPF1 siRNAs. **f-g** qRT-PCRanalysis of Flag-DDIT4 levels in SW1990 cells expressing Flag-DDIT4 with theDDIT4 CDS-3'UTR or DDIT4 CDS and treated with control vector or UPF1 plasmid.The sequence of the forwardprimer corresponds to the 3×Flag vector. **h-i **WB analysis of Flag-DDIT4levels in SW1990 cells expressing Flag-DDIT4 with the DDIT4 CDS-3'UTR or DDIT4CDS and treated with the control vector or UPF1 plasmid. **j **The levels of Ki67, Caspase-3,DDIT4 and p-mTOR were assessed in different groups using IHC. Dataare presented as the means ± SD of three independent experiments (*, *P* <0.05; **, *P*<0.01).

**Additional file 4.** Supplementary materials.

## Data Availability

The datasets used and/or analyzed during the current study are available from the corresponding author on reasonable request.

## Abbreviations

PDX: Patient-derived xenograft
GEM: Gemcitabine
CSC: Cancer stem cell
m^6^A: N^6^-methyladenosine
PDAC: Pancreatic ductal adenocarcinoma
DDIT4/ REDD1: DNA damage-inducible transcript 4
HuR: Human antigen R
ATCC: American Type Culture Collection
FBS: Fetal bovine serum
qRT-PCR: Quantitative real-time polymerase chain reaction
cDNA: Complementary DNA
IHC: Immunohistochemistry
IP: Immunoprecipitation
TCGA: The Cancer Genome Atlas
GTEx: Genotype-tissue expression
OS: Overall survival
DFS: Disease-free survival
FACS: Fluorescence-activated cell sorting
GO: Gene ontology
GSEA: Gene set enrichment analysis
NMD: Nonsense-mediated mRNA decay
