# Supplementary figures and images for: The m6A demethylase ALKBH5-mediated upregulation of DDIT4-AS1 maintains pancreatic cancer stemness and suppresses chemosensitivity by activating the mTOR pathway
Source: Mol Cancer. 2022 Sep 2;21:174. doi: 10.1186/s12943-022-01647-0 (PMC9438157; doi:10.1186/s12943-022-01647-0)

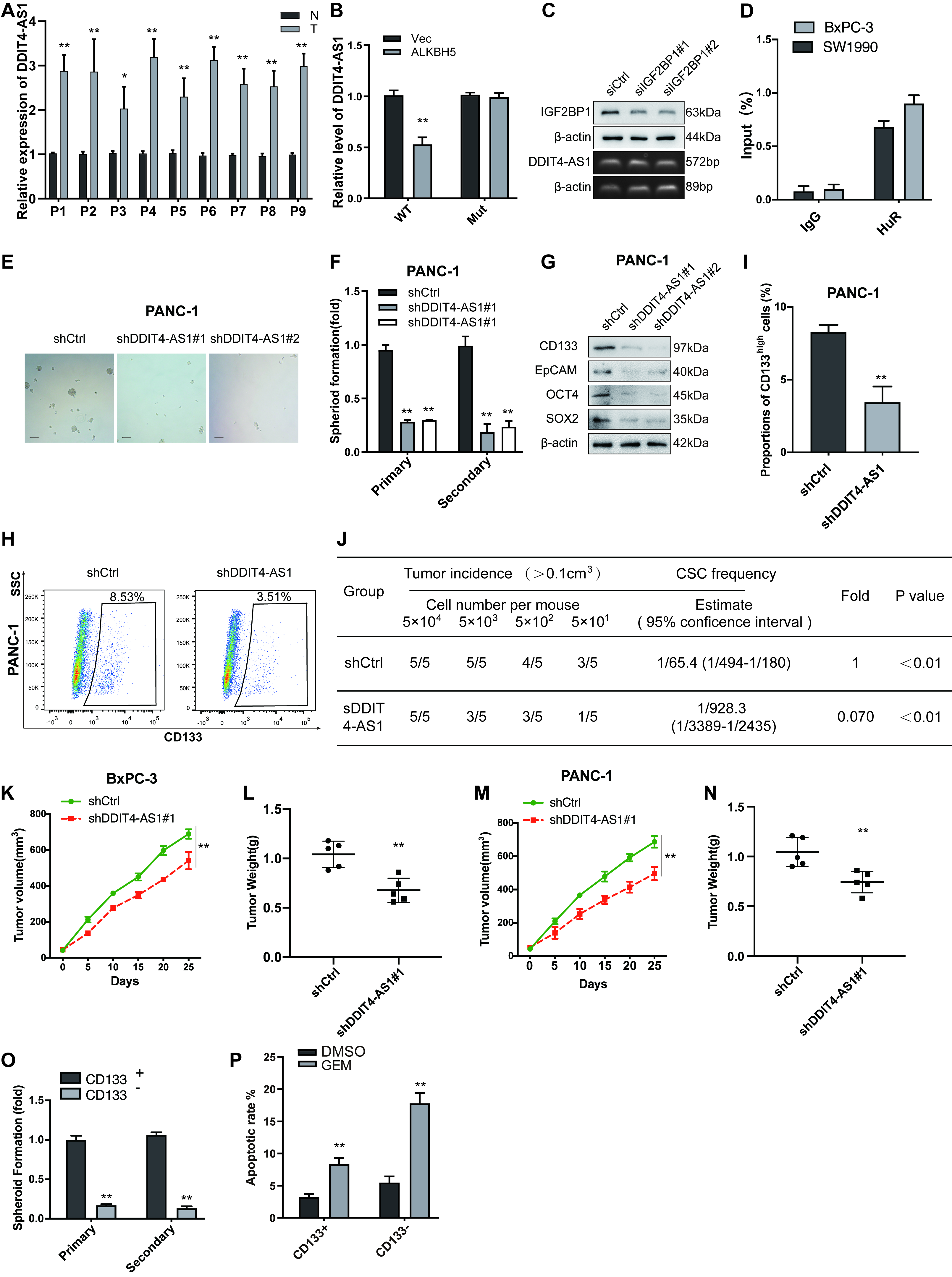

Supplement: Supplementary file 1 — Additional file 1: Figure S1. aThe RNA levels of DDIT4-AS1 in 9 pairedhuman PDAC tissues and normal adjacent tissues. b The relative RNAlevels of DDIT4-AS1 in BxPC-3cells cotransfected with the ALKBH5overexpression plasmid and DDIT4-AS1-WT or DDIT4-AS1-Mut. cWB and RT-PCRanalyses of IGF2BP1 and DDIT4-AS1 levels in BxPC-3 cells transfectedwith IGF2BP1 siRNAs. d qRT-PCR was used to determine the level of DDIT4-AS1 immunoprecipitatedby the anti-HuR antibody. e-f Representative images of sphere formation induced by thetransfection of shDDIT4-AS1 into PANC-1cells. The surviving colonies were measured by calculating the fold changes. g The expressionlevels of CSC markers were examined in DDIT4-AS1-silenced PANC-1 cells using Western blotting. h-i Flow cytometrywas performed to assess the percentage of CD133high cells among PANC-1 cells withDDIT4-AS1 depletion. jBxPC-3cells with or without DDIT4-AS1 depletion were injected into the subcutaneoustissues of nude mice at a density of 5 × 10^4^, 5 × 10^3^, 5× 10^2^ or 5 × 101 cells per mouse. The number of mice thathad developed tumours was counted. The frequency of CSCswas calculated using ELDA software. k-l Growth curves and tumour weight of nudemice after BxPC-3 cells with or without DDIT4-AS1 depletion wereinjected. m-n Growth curves and tumour weight of nudemice after PANC-1 cells with or without DDIT4-AS1 depletion wereinjected. o Sphere formation of sorted CD133+ and CD133-BxPC-3 cells. The spheroid formation were measured bycalculating the fold changes (the number of tumour spheres in theDDIT4-AS1-silenced PANC-1 cells relative tothat in ctrl cells). pSorted CD133+ cells and CD133- cells were treated withGEM. The apoptotic rate was detected using flow cytometry. Data are presented asthe means ± SD of three independent experiments (*, P < 0.05; **,P<0.01). [file 12943_2022_1647_MOESM1_ESM.tif]

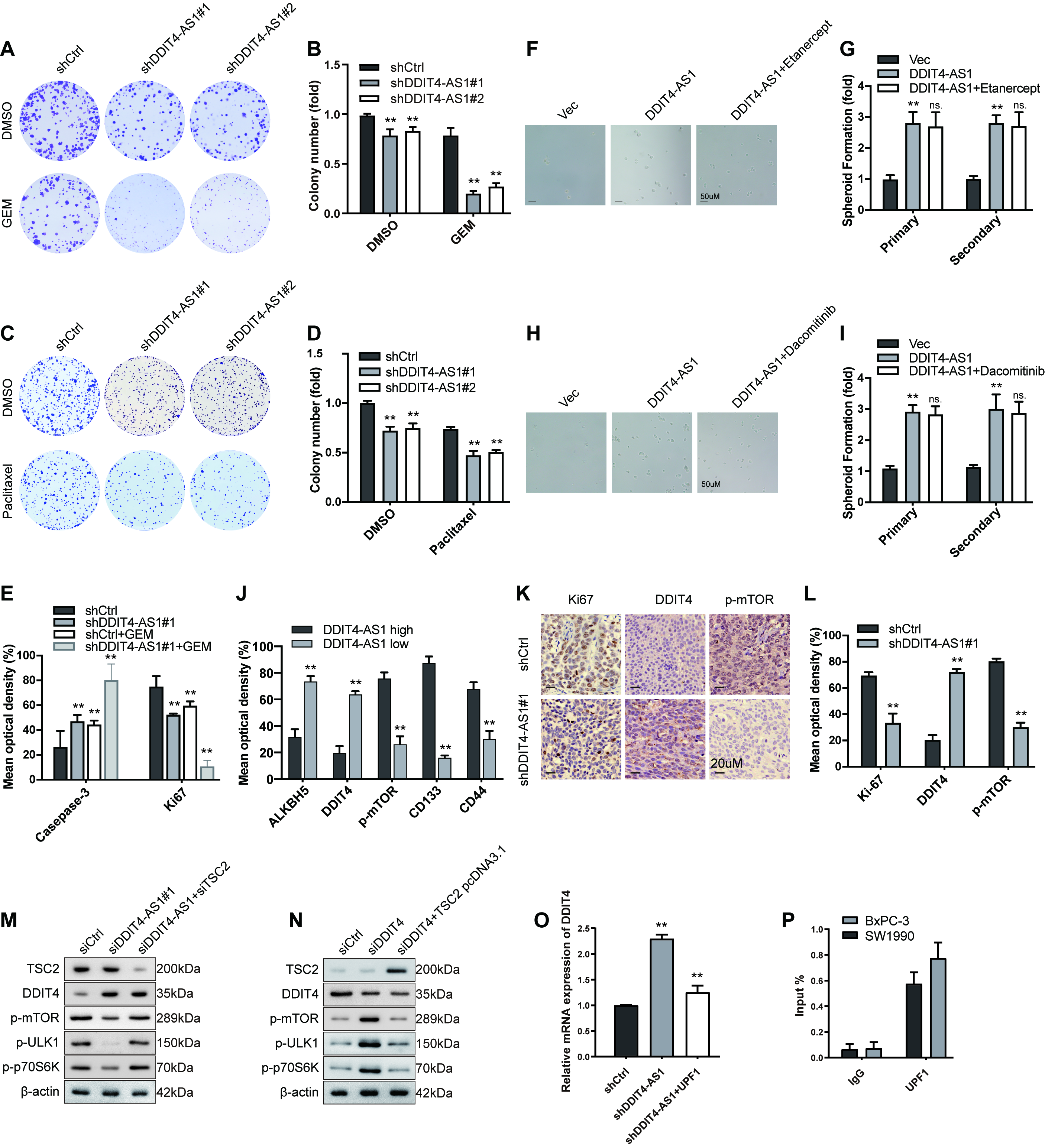

Supplement: Supplementary file 2 — Additional file 2: Figure S2. a-bColony formation by shDDIT4-AS1-silenced PANC-1cells after treatment with 10nM GEM. c-d Colony formation byshDDIT4-AS1-silenced BxPC-3 cells aftertreatment with 5uM Paclitaxel. e The nudemice were injected of BxPC-3 cells with orwithout DDIT4-AS1 depletion and treated with 60 mg/kg GEM twice a week. TheKi67 and Caspase-3 expression were evaluated by IHC staining. f-gRepresentative images of sphere formation in DDIT4-AS1-depleted BxPC-3 cells with or without etanercept treatment. Thespheroid formation was measured by calculating the fold changes. h-iRepresentative images of sphere formation in DDIT4-AS1-depleted BxPC-3 cells with or without dacomitinib treatment.The spheroid formation was measured by calculating the fold changes. j IHCstaining showing the expression of ALKBH5, DDIT4, p-mTOR, CD133 and CD44 inPDAC tissues with higher or lower levels of DDIT4-AS1 formedby shDDIT4-AS1 or control cells. k-lThe levels of Ki67, DDIT4 and p-mTOR were assessed in the shCtrl and shDDIT4-AS1#1 groups using IHC. m-n WB analysis of TSC2, DDIT4 andmTOR pathway-related proteins inDDIT4-AS1-depleted BxPC-3 cells transfectedwith TSC2 siRNA and DDIT4-depleted SW1990 cells transfected with TSC2 overexpressionplasmid. oThelevel of DDIT4 mRNA was examined in DDIT4-AS1-knockdown BxPC-3 cells with or without UPF1 overexpression using qRT-PCR.p qRT-PCR was used to determine the level of DDIT4-AS1immunoprecipitated by the anti-UPF1 antibody. Data are presented as the means ±SD of three independent experiments (*, P < 0.05; **, P<0.01). [file 12943_2022_1647_MOESM2_ESM.tif]

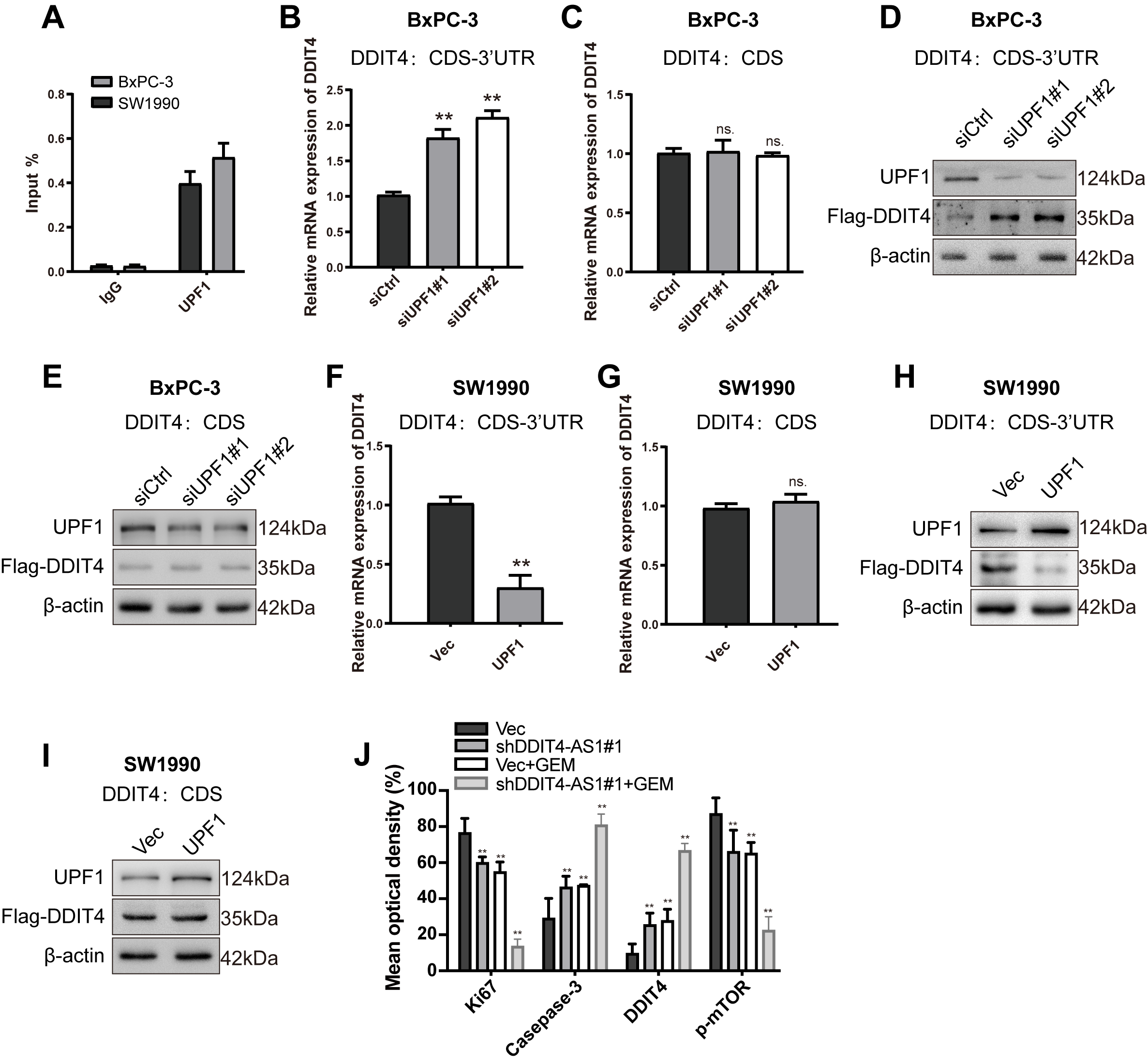

Supplement: Supplementary file 3 — Additional file 3: Figure S3. aqRT-PCR was performed to determine the level of the DDIT4 mRNAimmunoprecipitated by the anti-UPF1 antibody. b-c qRT-PCR analysis ofFlag-DDIT4 levels in BxPC-3cells expressing Flag-DDIT4 with the DDIT4 CDS-3'UTR or DDIT4 CDS and treatedwith control or UPF1 siRNAs. The sequence of the forwardprimer corresponds to the 3×Flag vector. d-e WB analysis of Flag-DDIT4levels in BxPC-3 cells expressing Flag-DDIT4 with the DDIT4 CDS-3'UTRor DDIT4 CDS and treated with control or UPF1 siRNAs. f-g qRT-PCRanalysis of Flag-DDIT4 levels in SW1990 cells expressing Flag-DDIT4 with theDDIT4 CDS-3'UTR or DDIT4 CDS and treated with control vector or UPF1 plasmid.The sequence of the forwardprimer corresponds to the 3×Flag vector. h-i WB analysis of Flag-DDIT4levels in SW1990 cells expressing Flag-DDIT4 with the DDIT4 CDS-3'UTR or DDIT4CDS and treated with the control vector or UPF1 plasmid. j The levels of Ki67, Caspase-3,DDIT4 and p-mTOR were assessed in different groups using IHC. Dataare presented as the means ± SD of three independent experiments (*, P <0.05; **, P<0.01). [file 12943_2022_1647_MOESM3_ESM.tif]
