# Supplementary material for: The m6A demethylase ALKBH5-mediated upregulation of DDIT4-AS1 maintains pancreatic cancer stemness and suppresses chemosensitivity by activating the mTOR pathway
Source: Mol Cancer. 2022 Sep 2;21:174. doi: 10.1186/s12943-022-01647-0 (PMC9438157; doi:10.1186/s12943-022-01647-0)
Supplement: Supplementary file 4 — Additional file 4. Supplementary materials. [file 12943_2022_1647_MOESM4_ESM.docx]

**1. Primer sequences**

| Gene | Forward (5′–3′) | Reverse (5′–3′) |
| --- | --- | --- |
| DDIT4-AS1 | CTACAACAGGTCATAACAAAAAT | ATGAAACAAAGGCTTAGGG |
| CD133 | ACACTACCAAGGACAAGGCG | TCTCCAACGCCTCTTTGGTC |
| EpCAM | GTCTGTGAAAACTACAAGCTGG | CAGTATTTTGTGCACCAACTGA |
| OCT4 | CAAAGCAGAAACCCTCGTGC | AACCACACTCGGACCACATC |
| SOX-2 | AGGATAAGTACACGCTGCCC | TTCATGTGCGCGTAACTGTC |
| DDIT4 | CCACCTCCTCTTCGCCCTC | AGCCACTGTTGCTGCTGTCC |
| β-actin | CCTTCCTGGGCATGGAGTC | TGATCTTCATTGTGCTGGGTG |
| FLAG-P1 | CCGAGTTCGAGTTCACCGAC | AACTGGCTGGTCTTGGCTAC |
| FLAG-P2 | CATAATTTGGTTAAGAGACATGCGG | CAGGGACCTTGATGACGTGG |
| FLAG-P3 | CCTCGGTGTCTGGCTACATC | ACGGCATAAACCTGGGAGTG |
| FLAG-P4 | CTCATGCAGTTCAGCAAGCC | TGCCAATCTGGTCATGGGTC |
| FLAG-P5 | AGCTCGCAGACTCTCACTTTC | GCGTCTGGCTAGGAAGAGTA |

**2. Antibodies**

| Antibody | Company | Cat. | WB | IHC |
| --- | --- | --- | --- | --- |
| ALKBH5 | Abcam | Ab195377 | 1;5000 | 1;50 |
| HuR | Abcam | Ab200342 | 1;10000 | 1;50 |
| β-actin | Abcam | Ab8226 | 1;20000 | 1;200 |
| CD133 | Abcam | Ab222782 | 1;10000 | 1;50 |
| EpCAM | Abcam | Ab223582 | 1;5000 | 1;50 |
| OCT4 | Cell signaling technology | #2750 | 1;2000 | 1;200 |
| SOX2 | Cell signaling technology | #2748 | 1;5000 | 1;200 |
| Ki67 | Abcam | Ab15580 | 1;20000 | 1;500 |
| Caspase-3 | Cell signaling technology | #14220 | 1;5000 | 1;50 |
| mTOR | Abcam | Ab2833 | 1;5000 | 1;50 |
| p-mTOR | Abcam | Ab232486 | 1;2000 | 1;50 |
| p-ULK1 | Abcam | Ab229909 | 1;2000 | 1;100 |
| p-P70S6K | Affinity Biosciences | #AF3228 | 1;5000 | 1;50 |
| DDIT4 | Abcam | Ab191871 | 1;5000 | 1;200 |
| CD44 | Affinity Biosciences | #DF6392 | 1;2000 | 1;100 |
| UPF1 | Abcam | Ab109363 | 1;10000 | 1;250 |
| SMG5 | Abcam | Ab129107 | 1;1000 | 1;100 |
| PP2A | Abcam | Ab32104 | 1;5000 | 1;50 |
| GAPDH | Proteintech | 60004-1-Ig | 1:20000 | 1;100 |
| TSC2 | Abcam | ab52936 | 1;20000 | 1;100 |
